# Effect of specific non-pharmaceutical intervention policies on SARS-CoV-2 transmission in the counties of the United States

**DOI:** 10.1038/s41467-021-23865-8

**Published:** 2021-06-11

**Authors:** Bingyi Yang, Angkana T. Huang, Bernardo Garcia-Carreras, William E. Hart, Andrea Staid, Matt D. T. Hitchings, Elizabeth C. Lee, Chanelle J. Howe, Kyra H. Grantz, Amy Wesolowksi, Joseph Chadi Lemaitre, Susan Rattigan, Carlos Moreno, Brooke A. Borgert, Celeste Dale, Nicole Quigley, Andrew Cummings, Alizée McLorg, Kaelene LoMonaco, Sarah Schlossberg, Drew Barron-Kraus, Harrison Shrock, UFCOVID Interventions Team, Stephanie Khoury, Meenal Indra, Hung-Leong Yau, Ben Cummings, Peter Giannas, Martha-Grace McLean, Ken Hubbard, Camazia Saunders, Caroline Weldon, Caroline Phillips, David Rosenbaum, Dianelys Tabla, Justin Lessler, Carl D. Laird, Derek A. T. Cummings

**Affiliations:** 1 Department of Biology, University of Florida, Gainesville, FL USA; 2 Emerging Pathogens Institute, University of Florida, Gainesville, FL USA; 3 Sandia National Laboratories, Albuquerque, NM USA; 4 Department of Epidemiology, Johns Hopkins Bloomberg School of Public Health, Baltimore, MD USA; 5 Department of Epidemiology, Brown University School of Public Health, Providence, RI USA; 6 Department of Civil and Environmental Engineering, École Polytechnique Fédérale de Lausanne, Lausanne, Switzerland; 7 Department of Mathematics, Syracuse University, Syracuse, NY USA; 8 Department of Public Health, Syracuse University, Syracuse, NY USA; 9 Tulane University, New Orleans, LA USA; 10 Department of Health Science, University of Vermont, Burlington, VM USA; 11 Northwestern University, Chicago, IL USA; 12 University of Texas Medical Branch, Galveston, Texas USA

**Keywords:** Computational models, SARS-CoV-2, Viral infection, Epidemiology

## Abstract

Non-pharmaceutical interventions (NPIs) remain the only widely available tool for controlling the ongoing SARS-CoV-2 pandemic. We estimated weekly values of the effective basic reproductive number (R_eff_) using a mechanistic metapopulation model and associated these with county-level characteristics and NPIs in the United States (US). Interventions that included school and leisure activities closure and nursing home visiting bans were all associated with a median R_eff_ below 1 when combined with either stay at home orders (median R_eff_ 0.97, 95% confidence interval (CI) 0.58–1.39) or face masks (median R_eff_ 0.97, 95% CI 0.58–1.39). While direct causal effects of interventions remain unclear, our results suggest that relaxation of some NPIs will need to be counterbalanced by continuation and/or implementation of others.

## Abstract (web summary)

Disentangling the impacts of non-pharmaceutical interventions on COVID-19 transmission is challenging as they have been used in different combinations across time and space. This study shows that, early in the epidemic, school/daycare closures and stopping nursing home visits were associated with the biggest reduction in transmission in the United States.

## Introduction

**Fig. 1:**
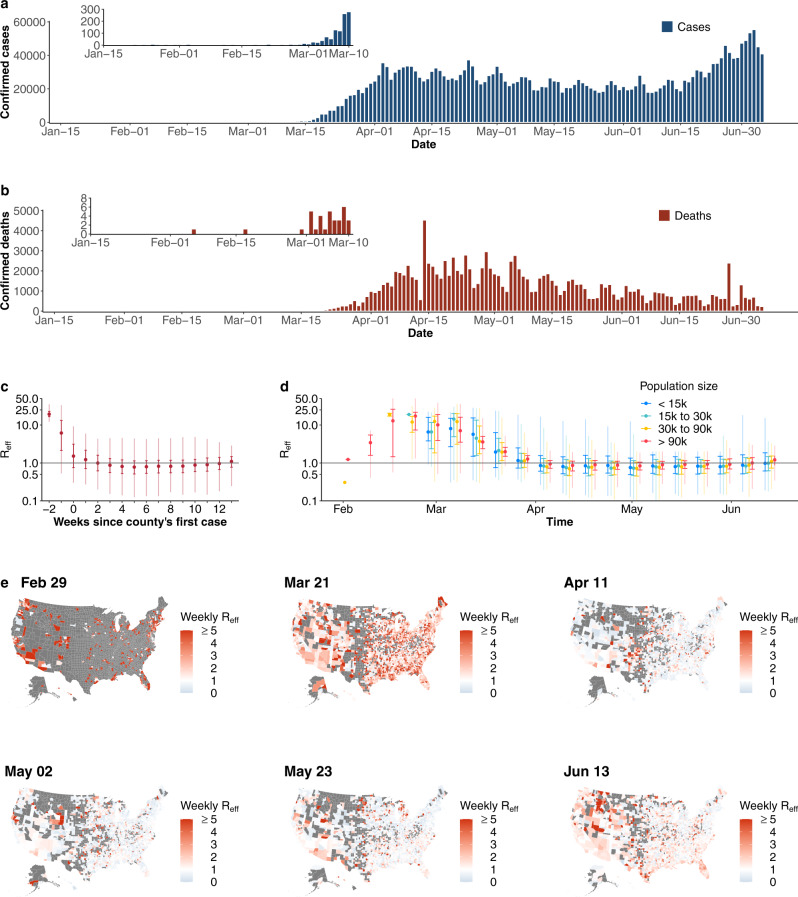
Confirmed COVID-19 cases and reproduction numbers (*R*_eff_) in the United States. **a** Daily confirmed COVID-19 cases. **b** Daily confirmed COVID-19 deaths. **c** Distribution of *R*_eff_ by weeks since the county’s first reported case (*n* = 36,737 county-weeks). Gray horizontal line indicates the threshold of *R*_eff_ = 1 (same as in **c**). Medians (points), interquartiles (dark vertical lines) and 95% percentiles (light vertical lines) are shown (same as in **d**). **d** Temporal distribution of *R*_eff_ stratified by county population size. County population size was classified into four groups, i.e., <15,000 (blue, *n* = 7656 county-weeks), 15,000–30,000 (green, *n* = 8139 county-weeks), 30,000–90,000 (orange, *n* = 10,929 county-weeks) and >90,000 (red, *n* = 10,013 county-weeks). **e** Map of county *R*_eff_ for representative weeks. Weeks were selected with a 3-week interval from the last week when *R*_*e*ff_ were available. Gray indicates no data available.

Months after the emergence of SARS-CoV-2 and its subsequent pandemic spread, widespread transmission continues. The United States (US) has been particularly affected, although the burden of disease has been geographically heterogeneous. While Northeastern states like New York, New Jersey, and Massachusetts were hardest hit when the virus first arrived in the US from March to April, 2020, states like Texas, Florida, Arizona, and California, which had previously avoided substantial disease burden, experienced rapidly growing cases between June and August (Fig. 1a)^1^. Many factors likely contribute to spatial and temporal heterogeneity in COVID-19 incidence, including socio-demographic characteristics, the frequency of importation of infections, and the local use and timing of non-pharmaceutical interventions (NPIs)^2–5^.

While there is a continuing need to control SARS-CoV-2 transmission, prolonged social and economic strains push the limits of population compliance. Hence, it is critical to identify targeted and effective strategies for disease control. Variation in NPI application and timing across US counties provides an opportunity to compare the effectiveness of interventions in reducing transmission, but these effects are difficult to estimate directly due to spatial and temporal variation in transmission and an imperfect and ever-changing surveillance system. We hypothesized that by using multiple data streams and focusing on estimates of transmissibility rather than raw counts of cases or deaths, some of these challenges may be overcome.

Here, we estimated the transmissibility of SARS-CoV-2 in 3035 (out of 3142, 97%) US counties using a mechanistic meta-population model that incorporates spatial coupling of transmission between counties to estimate weekly effective basic reproductive numbers (i.e., *R*_eff_, the reproductive number adjusted for changes due to factors other than population susceptibility, such as social distancing) from confirmed cases and deaths from January 21 to July 5, 2020. We associated these *R*_eff_ estimates with NPIs and county-level demographics, while accounting for, temporal variation, autocorrelation and uncertainty in our estimates. We aimed to determine which NPIs have been most effective so far to inform future implementations.

## Results

A total of 2,846,249 COVID-19 cases and 128,391 deaths were reported in the US as of July 5, 2020 (Fig. 1a). Cases first appeared in coastal counties with large populations in late-January and were reported in 68% of US counties by March 31. Weekly *R*_eff_ fit to confirmed cases (Fig. 1b, c) suggests that counties with larger population sizes (>90,000) experienced earlier and more efficient transmission (i.e. greater *R*_eff_) (median *R*_eff_ 2.6, interquartile range (IQR) 1.5–6.5 in the weeks before April) (Fig. 1c, d; Supplementary Fig. 1). Later in the epidemic *R*_eff_ dropped in these large counties (median *R*_eff_ of 0.8, IQR 0.7–1.0 in the week ending May 2), and though *R*_eff_ was similar in small counties, some had appreciably higher transmission (median *R*_eff_ of 0.9, IQR 0.6–1.8) (Fig. 1c, d).

**Fig. 2:**
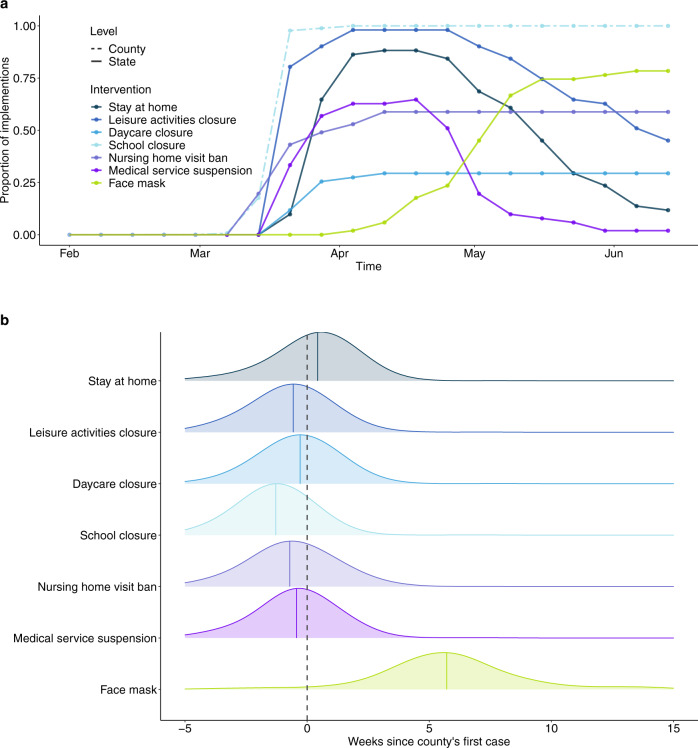
Temporal distributions of non-pharmaceutical interventions. **a** Time series of the proportion of states and counties where interventions were implemented. The color denotes the non-pharmaceutical intervention at county- (dashed) or state- (solid) level. **b** Distribution of time differences between intervention times and the occurrence of the county’s first confirmed case. Colored, solid lines indicate the median difference times of each intervention. Dashed vertical line indicates the week when the county reported its first case.

We compiled detailed data on the timing of state level NPIs policies (indicated by specific state-wide directives/orders^6^ and grouped correlated NPIs according to hierarchical clustering results; details see Supplementary Fig. 2 and Table 1) and county-level data on school closure. Enactment of NPIs started in early March and peaked the week ending April 11, by which time 100% of counties had closed public schools; and, of states, 98% had closed leisure activities (restaurants, gyms, and movie theaters), 88% had stay at home orders, 63% had suspended medical services, 59% had banned nursing home visits and 29% had closed daycares (Fig. 2a and Supplementary Fig. 3). In most counties, the majority of interventions were implemented before a county had its first case; with school closure coming earliest (median 1.4 weeks before first case IQR 2.6–0.6 weeks) (Fig. 2b). The one exception is face masking orders which were initiated on average 5.7 weeks (IQR 4.1–7.0 weeks) after the first case (Fig. 2b). Many locations started to gradually lift control measures in mid-April, particularly medical service suspensions (remained in only one state as of June 13 (2%)), stay at home orders (12%) and leisure activities closures (45%) (Fig. 2a and Supplementary Fig. 3). At time of analysis no county had lifted school closure.

In March, the average *R*_eff_ in each state was consistently high across the country (median 4.8, IQR 4.1–5.3), but transmission had reduced sharply by May (median 1.2, IQR 0.9–1.5). However, these results mask substantial variation within states at all points in the epidemic (mean intra-state coefficient of variation (CV) 1.39, IQR 1.28–1.44 in March, and 1.16, IQR 0.78–1.66 in May) (Supplementary Fig. 4). Using generalized estimating equations (GEEs) to account for the temporal autocorrelation of *R*_eff_ (Supplementary Fig. 5), we estimated the associations between county-level intervention policies and log-transformed *R*_eff_ adjusting for log-population size, proportion of individuals in poverty, median household income and other county-level covariates (Supplementary Tables 2–4; see Methods for description of all models considered). Transmission was consistently higher in counties with greater population sizes (21% increase in *R*_eff_ per 1000 increase in population size, 95% CI 13–29%) and those with a higher proportion of people without college educations (5% per 10% increase, 95% CI 3–6%), while transmission was consistently lower in counties with higher proportions of white individuals (2.6% per 10% decrease in the proportion, 95% CI 2.0–3.2%) and those with a lower median age (0.6% per 1 year decrease in median age, 95% CI 0.3–0.9%).

**Fig. 3:**
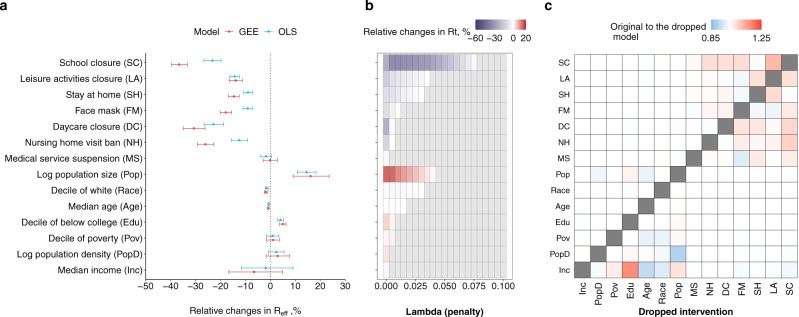
Associations between non-pharmaceutical interventions (NPIs) and county-level characteristics on transmission. **a** Associations between NPIs and county-level characteristics estimated from the main model (*n* = 31,072 county-weeks). Models were fitted with both generalized estimating equations (GEEs, red) and ordinary least squares (OLS, blue) models. Data are presented as mean and 95% confidence interval. The order in *y*-axis (same for **c**) is according to the importance of covariates in explaining the variances shown in **b**. **b** The importance of covariates in explaining the variances. Main models that were formulated for OLS models and fitted to least absolute shrinkage and selection operator (LASSO) with increasing parsimony. **c** Changes in the estimated effects when each covariate is dropped in the main OLS model.

We found that transmission had the strongest association with school closure (37% reduction in *R*_eff_, 95% CI 33–40%), followed by daycare closure (31%, 95% CI 26–35%) and banning nursing home visits (26%, 95% CI 23–29%) (Fig. 3a; main model in Supplementary Tables 2, 3). Stay at home orders were associated with a 15% reduction in *R*_eff_ (95% CI 13–17%) while face-mask orders were associated with an 18% reduction (95% CI 16–20%). Leisure activities closure were associated with a 14% (95% CI 11–17%) reduction in *R*_eff_, while with a 5.0% (95% CI −1.9 to 12.4%) increase when lifted in the sensitivity analysis (Supplementary Fig. 6). OLS models that included a lag-term to account for autocorrelation (termed the OLS model, an alternative approach to GEE) yielded similar regression coefficients (Fig. 3a and Supplementary Table 5). To ensure the contribution of interventions in reducing *R*_eff_ when prioritizing parsimony, we fitted LASSO regression on the OLS model across a range of penalties for model complexity and found that school closure was the intervention most consistently associated with reductions in *R*_eff_ (Fig. 3b). Including information on NPIs improved model performance across multiple metrics (Supplementary Tables 3, 4) (see Methods).

Implementation of interventions was highly temporally correlated both between and within counties (Fig. 2, Supplementary Figs. 3, 4), presenting challenges to estimating independent associations. For example, school closures occurred at the same time in many countries, with closure only reported in three unique weeks making separation of the effect of school closure from the effect of week of year impossible (including week of year as a categorical covariate eliminates the observed effect; Supplementary Fig. 7). However, whether causation, coincidence or confounding, reduction in *R*_eff_ occurred for some reasons that cannot be explained purely by other events that happened at the same time (i.e., state of emergency and CDC guidelines; Supplementary Figs. 8, 9); hence, we performed several analyses to better disentangle the observed associations with NPIs.

Univariate and multivariate models for each NPI showed similar relative associations (Supplementary Fig. 10) as did models that included an effect for time since the first case or first 10 cases in a county (Supplementary Figs. 11, 12). To assess the impact of collinearities, we reran the main model holding out each NPI one at a time. The largest changes in estimated coefficients were seen when dropping school closure, which substantially impacted the estimated association of *R*_eff_ with remaining coefficients (e.g., the coefficient of banning nursing home visits indicated a 6.5% larger reduction in models without school closure) (Fig. 3c). To further ensure the observed associations between NPIs and reductions in *R*_eff_ were not merely the result of spurious associations between changes in *R*_eff_ and changes in NPI, we permuted data on NPIs across counties (Supplementary Figs. 13 and 14a, b) and within counties (Supplementary Figs. 13 and 14c, d), respectively and found all associations to be substantially dampened or eliminated.

People changed their behavior in response to the pandemic, whether due to policy or personal choice. An important behavior that impacts SARS-CoV-2 transmission is travel to and social contact in different settings. Data on workplace presence relative to pre-pandemic periods were available on Google users^7^ and, unlike other measures of movement in these data, were available for the nearly all (98%) of the county-weeks in our analysis. Across the US, large changes in workplace presentation occurred in March (Supplementary Fig. 15). We explored potential mediation/confounding by workplace presence in each county, as we believe NPIs may achieve their effects through reduction in contacts in the particular venue of interest by the resultant reduction in workplace presentation. We found that, at least in part, the relationship with a number of NPIs including school closure (32% [95% CI 28%, 34%] of total effect explained), leisure activities (65% [95% CI 59%, 71%] and stay at home orders (100% [95% CI 94%, 109%]) were mediated and/or confounded by workplace presence (Supplementary Figs. 16, 17).

**Fig. 4:**
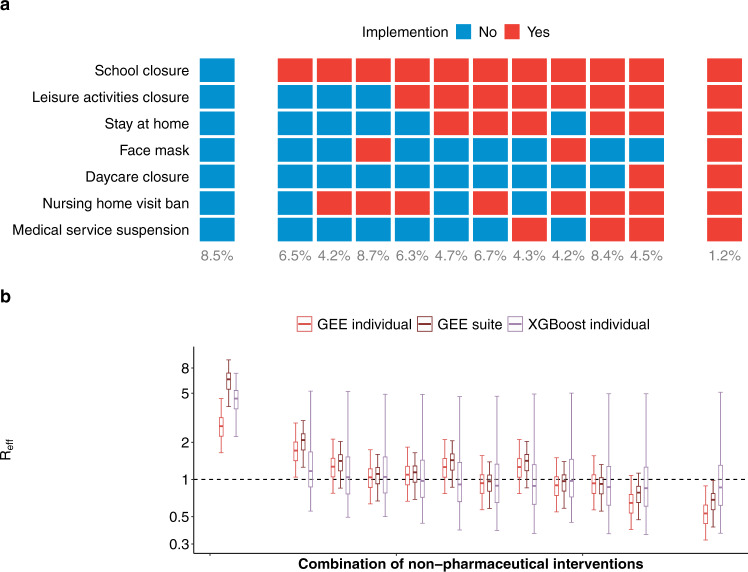
Prediction of reproduction numbers (*R*_eff_) associated with different non-pharmaceutical interventions (NPIs) suites. **a** Non-pharmaceutical interventions (NPIs) suites: no intervention (left column), all interventions (right column) and the ten most frequently used NPI suites in counties in the United States (middle columns; ordered by number of NPIs implemented (red; otherwise, blue) in each suite). Numbers below the columns are percentages of county-weeks in the study in which the suites were active. **b** *R*_eff_ with different NPI suites (*n* = 34,778 county-weeks). Median (cross), interquartile range (boxes) and 95% quantile range (vertical lines) of *R*_eff_ were distributions of predictions from the main model fitted with generalized estimation equations (GEEs, red, Fig. 3a), GEEs with NPIs fitted as suites (brown), and boosted decision tree (XGBoost individual, purple). *X*-axes are NPI suites that were shown in **a**. Horizontal dashed line indicates the threshold of one. Data are presented as median, interquartile, and 95% quantiles.

No single intervention was implemented alone for a sustained period of time in the period of our study, and many combinations of interventions never appear (e.g., there are no cases where stay at home orders are in effect but school closure is not). Hence, we only observe the associations of reduction in *R*_eff_ and suites of interventions that may have complex interactions. We fit a GEE model to each of the unique suites of NPI utilized by counties (including no intervention) as categorical variables (Fig. 4). We also fit a boosted regression tree model that can account for complex interactions between interventions (see Methods). Estimates from these models were consistent with those calculated from our main model. Interventions that included school and leisure activity closure and nursing home visiting bans were all associated with an *R*_eff_ below one when combined with either stay at home orders (median *R*_*eff*_ 0.97, 95% CI 0.58–1.39) or face masks (median *R*_*eff*_ 0.97, 95% CI 0.58–1.39) (Fig. 4). Inclusion of more interventions further reduced *R*_eff_, with a minimum median *R*_*eff*_ of 0.50 (0.30, 0.86) when all interventions are in place (Fig. 4, Supplementary Table 6).

Multiple sensitivity analyses found broadly similar estimates of associations of NPI with *R*_eff_, these included: replacing county-level school closure with state-level (Supplementary Fig. 18); subsetting our analysis to exclude highly uncertain *R*_eff_ estimates from early in the pandemic (Supplementary Fig. 19); allowing interventions to have an ongoing impact after they are lifted (Supplementary Fig. 6); including spatial clustering of *R*_eff_ (Supplementary Figs. 20, 21);using alternative reconstruction methods (Supplementary Fig. 22); and using estimates derived from reported deaths (Supplementary Fig. 23). In the last analysis, we did observe reduced effects of most NPIs and a larger effect of school closure; though results were otherwise qualitatively the same. In addition, sensitivity results suggested that our assumptions on reporting rate were less likely to affect our estimations on *R*_eff_ (Supplementary Fig. 24 and Table 7).

## Discussion

A strength of our approach is the detailed data on NPIs that we compiled as well as the robust estimation of transmissibility using a mechanistic meta-population model. We found that six of seven NPIs examined (except for medical service suspension) were associated with reductions in *R*_eff_, with school closure being associated with the greatest reduction. Although these associations were robust to unconsidered confounders (e.g., county-level characteristics, Supplementary Fig. 24), we can only speculate about the mechanistic pathways by which any of these policies may have caused reductions in transmission. School closures, for example, may have had substantial impacts on the social interactions of nonschool-aged individuals as parents and workplaces adapt to accommodate changes in children’s schedules, as suggested by our mobility analysis. It may also be a leading indicator of community attitudes about transmission. Our estimates indicate that four interventions were necessary, on average, to reduce *R*_eff_ below one, and that even with seven interventions, reproductive numbers remained above 0.65 on average (Fig. 4). However, we note that our estimates do not include the effect of immunity due to substantial infection in many areas of the US, which will cause additional reductions in *R*_eff_. Overall, even when the greatest number of intervention policies were in place (only observed in 1.2% of county-weeks; Fig. 4a), we never saw reductions as large as those seen in Asia and parts of Europe, where reproductive rates fell as low as 0.44^8^. Whether this is the result of poor compliance, structural factors, or states easing restrictions before they could have their full effect remains unknown. However, even though reductions were not as large as those seen in other areas, stark and immediate reductions in reproduction numbers across the US coincided with the use of NPI.

We are not the first to attempt to estimate the potential impact of NPIs on transmission of SARS-CoV-2. Previous analyses agree with ours in several important dimensions, including the clear association of business closures, stay at home orders, and masking wearing with significant reductions in transmission^9–14^. Beyond the potential impact of NPIs, other studies identified population density as a risk factor for transmission^15^ while we found that county population size had a stronger association (Supplementary Table 8).

There remains debate on the role of school closure in reducing transmission. Consistent with our results, Brauner et al.^11^, Liu et al.^16^, and Auger et al.^2^ found significant associations between school closure and reduced transmission or incidence, while Li et al.^17^ reported increased transmissions after school reopening. However, others, e.g., Hsiang et al.^9^, have found no association between school closure and the rate of growth in cases. A recent study found in-person schooling associated with increased household risks of COVID-19^18^, suggesting future studies about the direct and indirect effects of school closures on transmissions could aid in designing mitigation measures. We never see an instance in the US where stay at home orders and other effective interventions are implemented without school closure by the time this study was conducted, so all other associations are measured in the presence of school closure.

The impact of school closures need not necessarily stem directly from reductions in direct infection by school aged individuals. The associated reductions in *R*_eff_ we observed suggest substantial confounding or indirect effects of school closure. The association between school closure and time spent in the workplace provides one possible mechanism of indirect effects, however others exist. Further, even if transmission is rare, schools bridge many communities, and can play an important role in facilitating epidemic spread by connecting these subpopulations. Regardless of why, the data and past experience show important associations between school closure and transmission which should not be dismissed when setting policy.

Aside from the correlation in timing of interventions, other factors may also challenge our inferences. Compliance with policies and lags between implementation and actions by individuals could obscure the associations between policies and transmission. School closure is unique in this regard among the NPI we considered as schools stayed closed with effectively 100% compliance over the period of our analysis. There may be confounders or mediators that were unmeasured or not included in our model. For instance, testing, contact tracing, and quarantine were found to be effective in other studies, but data were not available at the time we performed the analyses. Though we were able to obtain county-level data on school closures, we were limited to state level data on other interventions. Local changes that affected large populations within a state may lead to misclassification bias. Large spatial and temporal variation in the accuracy of surveillance for confirmed cases or deaths could induce spurious changes in *R*_eff_ that do not reflect true transmission^19^, however our conclusions were robust to the unobserved spatial-temporal clustering patterns of the data (Supplementary Fig. 21). In addition, using both cases and deaths for our inference helps mitigate this possibility. Many counties reported limited numbers of cases and/or deaths and thus infection dynamics could not be reconstructed. We assumed a stable distribution of delays between infection and the time of confirmation or death, though this could have varied over the course of the outbreak^20^. Not all behavioral change is captured by concrete policies (e.g., voluntary behavior in response to the locally reported cases, which can be displaced by the mandate orders^21^); however, our focus was on the possible impact of policies enacted by governments rather than actions taken by individuals in the absence of such policies.

Despite these limitations our analysis provides critical insight into how individual interventions, or at least commonly used suites of interventions, may affect the spread of SARS-CoV2. These estimates are critical as governments attempt to figure out how to respond to resurgent cases and look for responses that successfully control spread while allowing as much of normal economic and social life to continue as possible. We found lifting leisure activities (e.g., restaurants and gyms) were associated with increased *R*_eff_, indicating higher transmission risks in these settings^22,23^. We estimated less dramatic changes in *R*_eff_ associated with the removal of stay-at-home orders and medical service suspension (Supplementary Fig. 6). The fact that multiple NPIs were needed to observe *R*_eff_ below one, suggests that relaxation of some NPIs might need to be counterbalanced by continuation and/or implementation of others. Our point estimates of the relative contribution of each intervention provide some guidance in making these difficult decisions.

## Methods

### Data on COVID-19 cases and deaths

Laboratory-confirmed COVID-19 cases and deaths in the counties of the United States were retrieved from USAFacts (https://usafacts.org/visualizations/coronavirus-covid-19-spread-map/) on July 5, 2020. The data from USA Facts was used for all counties except for New Mexico, where we observed timing offsets in weekend reporting for a few, select dates and counties. Data from the NY Times (https://www.nytimes.com/article/coronavirus-county-data-us.html) did not have these issues and thus were used for New Mexico.

### Data on county-level demographic characters

We obtained data on county-level demographic characteristics (i.e. population size, population median age, land area, median annual income, number in poverty, number reported as white in race, and number with education below college) from the 2014 to 2018 American Community Survey, which were retrieved through tidycensus package version 0.9.9.2^24^ in R. Population density was calculated by dividing the population size over land area. Proportions of population in poverty, white in race, and education below college were derived by dividing the numbers with the total number of people surveyed for those characteristics.

### Data on non-pharmaceutical interventions (NPIs)

We obtained dates of policy announcements of closures of public schools (K-12) of each county by consulting the government websites of school district, county and state and local news sources. We used the earliest documented date as the county’s school closure date when there were multiple dates available for districts within that county. When closures were announced during or at the end of a planned school break, the date when schools were last in session was reported. We found school closure dates for 94.3% (2963 out of 3142) of counties, which were further included in our analyses.

We obtained dates of implementation and termination of the other NPIs at state-level from COVID-19 US State Policy Database (CUSP) on July 6, 2020^6^. Eleven interventions that were directly used to reduce transmissions were extracted from the dataset (Supplementary Table 1). Interventions were grouped if they were semantically similar and clustered in time (e.g., face mask mandated in the public and face mask mandated in businesses; Supplementary Fig. 2). When multiple dates of interventions and terminations were available for the grouped NPIs, we used the earliest documented date for implementations and the latest date for termination. An intervention is considered as implemented if the date of the corresponding *R*_eff_ was between its implementation and termination date.

### Data ethics

Google’s mobility data consists of aggregated, anonymized sets of data from users who have chosen to turn on the location history setting. Consent for use was obtained by Google when users chose to turn on the location history setting.

### Estimating reproduction number

We fit a mechanistic transmission model to confirmed cases of COVID-19 in each of the counties of the US. Separately, we also fit these same models to deaths due to COVID-19 in each county. We used spatially coupled SEIR models to represent the transmission of SARS-CoV-2 with separate metapopulations representing the population in each US county. We estimated the incidence of infection in each county based on confirmed count data (and separately deaths due to COVID-19) using the back-projection method of Becker et al^25^. For our deconvolution procedure, numbers of cases and deaths were upscaled by sampling from a negative binomial (with probability 1/10 and 1/200 respectively). Second, using delay distributions for cases and deaths (see the section of natural history of SARS-CoV-2 below), we applied the function backprojNP from package surveillance^26^ in R to back-project incidence. Finally, to account for right-truncation of incidence values, we followed Abbott et al.^27^ and sampled the estimated incidence to account for infections that had occurred but had not yet been reported or confirmed from a negative binomial distribution, where the probability was given by the respective cumulative delay distributions. The most recent 14 days were then removed. We constructed 100 stochastic realizations of this algorithm for each county. Using the resultant time series of daily infections in each county, we fit our mechanistic model with state variables S (susceptible to SARS-CoV-2), E (exposed, infected but not yet infectious), I (infectious), and R (recovered or deceased and no longer infectious) to each stochastic realization, which included three infectious components to allow the distribution of durations of infectiousness to approximate a gamma distribution^28^. The migrations between compartments were random samples from binomial distributions with probability of the calculated migration rate. A mobility matrix derived from US Census commuting data from pre-pandemic time periods was used to specify the fraction of commuters in each county and the fraction of time that those commuters spent in counties other than the ones they reside^28,29^. For susceptible non-commuters and the fraction of time when commuters spent in local counties, the force of infections (FoI) were the full FoI in the local counties, while for the fraction of time when commuters spent in other counties, the FoI were that from the other counties^28^. Transmission coefficients ($\beta$) were estimated based on least-square regression of the observed and estimated infections in each county-week. Estimates used 2 weeks of incident infections to derive each piecewise constant transmission coefficient in each week (with estimates assigned to the first week of the 2 weeks used to estimate infections). Models were developed using epi_inference (a software package to be released), with mathematical programming performed with Pyomo^30^ and solved by IPOPT (a large-scale nonlinear optimization tool)^31^.

In order to examine the impact of reporting rate on estimating *R*_eff_, we performed several sensitivity analyses by assuming different fixed reporting rates for cases (1/8 and 1/12) and deaths (1/160 and 1/240; Supplementary Table 7). We also ran an analysis with a time-varying case reporting probability, obtained by assuming a fixed case-fatality rate, and assuming that the ratio of reported cases to deaths indicated the probability of reporting a case. To do this, we backprojected reported cases and deaths per county using the aforementioned delay distributions, aggregated the numbers up to a national level, took their ratio, and fit a generalized additive model with smoothing over time. This ratio, together with the reporting probability for deaths, then gives the time-varying reporting probability for cases (Supplementary Fig. 25). We calculated the Spearman correlation between *R*_eff_ used for the main analysis with the new sets of estimates. We also calculated the proportion of county-weeks where the new estimated *R*_eff_ fell in the range of the *R*_eff_ that were estimated from 100 realizations in the main analysis.

### Natural history of SARS-CoV-2

Time delays from infection to confirmation among those cases that are confirmed was assumed to be log-normally distributed with mean of days (log(8) = 2.07 days). This assumption was derived from^32^ and assumed confirmation comes on average 1.5 days after attendance at a medical facility. We assumed a log-standard deviation of 0.3 of this distribution^33^. Time delays from infection to death among those that die was assumed to be log-normally distributed with log-mean 2.84 and log-standard deviation of 0.72 and assuming no competing risks^34,35^.

### Estimating effects of NPIs on transmission

In general, models that included different sets of covariates (i.e., autoregression of *R*_eff_, additional temporal marker for county-time, county-level demographic characteristics and NPIs; see details in Supplementary Tables 2, 3) were fitted to log_10_(*R*_eff_) with GEEs (geeglm of geepack package^36^) and autoregressive OLS mode, separately. We included AR(1) in GEEs and included log_10_(*R*_eff_) in the previous week in the OLS model to account for autoregression of *R*_eff_. The two models represent two different analytic assumptions regarding the temporal autocorrelation: the OLS model treats previous observations of *R*_eff_ as a predictor variable, which would not affect the estimated variance of effect sizes, while GEEs assume both point estimates and standard errors can be affected by the correlation structure. We weighted log_10_-*R*_eff_ with its inverse coefficient of variation across the above-mentioned 100 stochastic reconstructions. NPI were included as a time-varying covariate with the status of the NPI defined for each week of the analysis as either 1 if in use in that county in a particular week or 0 if not. Supplementary Table 2 describes the components of the main model and alternatives, base, the model with the minimum number of covariates that we considered, time, which included the covariates included in base, but adding time since the first case in each county as a categorical variable and time and interventions, which adds NPI to the time model.

In our main results, we interpreted the estimated effects of NPIs from the main model that was fitted with GEEs (Fig. 3a). Autoregression of *R*_eff_, county-level characteristics and NPIs were included in the main model (Supplementary Table 2). To examine the reduction of different combinations of NPIs on *R*_eff_ (Fig. 4), we calculated *R*_eff_ by combining effects for individual NPIs that were estimated from the main model. To further examine whether the effects of NPI suites were robust to interactions between NPIs, we (1) fitted another model with GEEs by including autoregression of *R*_eff_, county-level characteristics and NPIs suites (as categorical variables); and (2) fitted a model with XGBoost of individual NPIs (details described below). We then compared the predictions on the *R*_eff_ for NPIs suites from the GEE suite model and XGBoost model to those calculated from our main model (Fig. 4b).

To increase the interpretability, we presented the proportion of reduction in *R*_eff_ when a given NPI or NPI suite were implemented, which was calculated as $1-1{0}^{\beta }$ and $\beta$ is the estimated coefficient for individual NPI or NPI suite.

### Sensitivity analyses of effects of NPIs on transmission

#### LASSO

To examine the relative importance of NPIs in explaining variance of *R*_eff_ with increasing parsimony of the model, we performed LASSO (glmnet in glmnet package^37^) with the main model that was fitted with our OLS model. The estimated effects of NPIs from the OLS model are highly correlated with those estimated from GEEs (Fig. 3a). Effect sizes of NPIs were presented as the complexity penalizing hyperparameter ($\lambda$) was increased from 0.0 to 0.1 at intervals of 0.005 (Fig. 3b). Coefficients estimated when $\lambda$ is 0 (i.e., no penalty) are equivalent to the estimates from the OLS model shown in Fig. 3a.

#### Collinearity of covariates

We assessed the potential impact of colinearity of NPIs on our estimated associations of NPI with *R*_eff_ through two approaches: dropping one NPI at a time (dropped model hereafter) prior to fitting the model, and by fitting a single-intervention model. For the dropped model, we dropped individual covariates of NPIs and county characteristics one at a time and reran the main model that was fitted to the OLS model. We calculated the ratio of relative changes in *R*_eff_ that was estimated from the main analysis to that estimated from the dropped models (Fig. 3c). For the single-intervention model, we added one NPI into the base model at a time and compared the estimated effect of that NPI with the original effect size that was estimated from the main model. Single-intervention models were also fitted to GEEs and OLS models (Supplementary Fig. 10).

Impact of temporal trend of *R*_eff_ on estimating effect of NPIs. In competing NPIs against temporal markers, we further included a fixed-effect associated with county-time (i.e., time since a county had its first case) into the main model as a weekly discretized coefficient that was shared across all counties (Supplementary Tables 2, 3). Weeks since the first case in a county were computed for each data point and were included in the models as categorical variables, i.e., from 3 weeks before to 13 weeks after the county saw its first case. In addition, we used time since a county had its first 10 cases as a proxy of county-time to account for the uncertainty that may be associated with small outbreak sizes (Supplementary Fig. 12).

In order to examine whether the effect of NPIs estimated from our main models was not just capturing the declining trends of *R*_eff_ over time, we first permuted NPIs spatially (i.e., permuted the NPIs suites between counties) and temporally (i.e., permuted the time series of NPIs within counties) and refit the main model (Supplementary Figs. 13, 14). We performed the permutations tests 100 times. Next, we fitted our main GEEs and OLS models by adding an additional variable of state of emergency, which was highly correlated with school closures in time (Supplementary Fig. 8). Finally, we split the school closure variable that was included in the main models into the week when CDC guideline first issued (called week of CDC guideline issued) and the rest of the weeks when school was closed (called after CDC guideline issued), in order to examine whether the effect of school closure was due to omitted variables happened at the same time (Supplementary Fig. 9).

#### Impact of uncertainty from early transmission phase

*R*_eff_ estimates at the beginning of an outbreak are often challenged by large uncertainties. In order to examine the effects of these uncertainties on our estimated effects of NPIs, we fitted the main model to a subset of our data set, which only includes *R*_eff_ since 2 weeks after the county saw its first case.

#### Impact of uncertainties of standard errors on estimating effect of NPIs

To examine the effect of spatial correlation of *R*_eff_ on the estimated effect sizes, we refitted the main GEEs model by changing the correlation structure to adjust for county-level clustering and adding the log *R*_eff_ in previous week to account for temporal autocorrelation (Supplementary Fig. 20). To account for the robustness of our results to the potential spatial-temporal clustering, we assessed the cluster-robust standard error (fixest package) for the main OLS model, in which two-way clusters of county and week were calculated (Supplementary Fig. 21).

### Effects of NPIs relaxations on transmission

In order to look at the effects of relaxing NPIs on the transmission, we fitted the main model by further splitting the effect of NPIs into during the implementation (intervention on) and after the implementation was lifted (intervention off) (Supplementary Fig. 6). Relaxations were available for nonessential business, stay at home and medical service suspension. The rest of the covariates were the same as in the main model.

### Using cases from stochastic reconstruction as alternative data to estimate *R*_eff_

*R*_eff_ that were estimated from an alternative stochastic reconstruction method of COVID-19 cases were derived to assess the robustness of our statistical inference to using these data compared to confirmed cases (Supplementary Fig. 22). We then used the above-mentioned methods to estimate weekly *R*_eff_ from confirmed cases and refit the main models with GEEs and OLS model regression (Supplementary Fig. 22). In the alternative reconstruction method, we first reconstructed the daily number of reported cases through a resampling procedure to account for the uncertainties arising from incubation period and health seeking behaviors. We fitted a negative binomial distribution to cases in each sliding window of 14 days and resampled the daily number of reported cases from the fitted distribution. We then estimated the time profile of transmissions by stochastic reconstructing the number of individuals in each transmission compartments, assuming the daily number of cases followed a binomial distribution with the above-mentioned confirming rate and delay intervals between infection to report. Finally, we performed forward simulations with the reconstructed time profile of transmissions and the above-mentioned SEIR model using the epi_inference software, where the migration between compartments followed by a binomial distribution with mean of the computed probability^28^. 100 realizations were computed.

### Using deaths as alternative data to estimate *R*_eff_

*R*_eff_ that were estimated from the deconvoluted COVID-19 deaths were derived to assess the robustness of our statistical inference to using these data compared to confirmed cases. We therefore used the above-mentioned methods to estimate weekly *R*_eff_ from confirmed deaths and refit the main models with GEEs and OLS model (Supplementary Fig. 23). *R*_eff_ were estimated for 1840 out of 3142 counties (58.6%).

### Out-of-sample prediction

Models were fitted with each of the fifty states and District of Columbia held out as test sets. Prediction performances were measured using root mean squared error (RMSE), mean absolute scaled error (MASE), and coefficient of determination (*R*^2^). Fitting procedures for the OLS models were as described above.

#### Comparative model

We employed XGBoost^38^, a decision tree boosting package, to explore whether more predictive power can be gained through complex model structures. Optimal values of three main hyperparameters (fraction of covariates included in each boosting iteration, fraction of training data included per those iterations, and maximum tree depth) were determined through grid search; ranges (and grid intervals) were 0.3–0.9 (0.1), 0.3–0.9 (0.1), and 3–9 (1), respectively. Performances were evaluated under 10-fold cross-validation. Learning rate was conservatively set to 0.2 and the maximum number of iterations was capped at 200 with early stopping if RMSE does not improve after two iterations to avoid overfitting. The respective optimal values were 0.9, 0.3, and 6. We further optimized the maximum iterations cap when test sets were held out by spatial units; range of 50–200 at intervals of 25. Results reported were from the optimal iteration of 75 (which minimized RMSE).

### Effects of NPIs mediated by workplace attendance

Google Community Mobility Reports were downloaded on September 16, 2020^7^. Mean of daily percentage change of work commutes relative to the baseline of each county were computed for each weekly interval where we have *R*_eff_ estimates. We chose to focus on workplace presentation among candidate datasets available through Google as this was the only dataset that had less than 50% of county-weeks missing. Mediation analyses were conducted for each NPI separately. For each NPI, we fitted a full model to log_10_ transformed weekly *R*_eff_ and adjusted for county-level characteristics, the workplace attendance and the examined NPI. We then fitted a mediation model which regresses the workplace attendance on the examined NPI. The mediation analyses were conducted using R package mediation^39^.

### Reporting summary

Further information on research design is available in the Nature Research Reporting Summary linked to this article.

## Supplementary information

Supplementary Information

Reporting Summary

## Data Availability

We collected the closure time of public schools (K-12) of each county, which can be accessed at https://github.com/UF-IDD/US_County_Rt/blob/main/data/school.csv. Data on other state-level interventions were obtained from an external data set COVID-19 US State Policy Database (CUSP) on July 6, 2020 at https://docs.google.com/spreadsheets/u/1/d/1zu9qEWI8PsOI_i8nI_S29HDGHlIp2lfVMsGxpQ5tvAQ/edit#gid=973655443. Laboratory-confirmed COVID-19 cases and deaths in the counties of the United States were retrieved from USAFacts (https://usafacts.org/visualizations/coronavirus-covid-19-spread-map/) on July 5, 2020. Data on commuting between counties before the pandemic was obtained from Census Bureau (https://www.census.gov/topics/employment/commuting.html). Data on county-level characteristics were obtained using “tidycensus” package version 0.9.9.2 (https://cran.r-project.org/web/packages/tidycensus/index.html). The authors declare that all data generated or analyzed during this study are made available at https://github.com/UF-IDD/US_County_Rt.

## Code availability

The authors declare that all codes for analyzing the data are made available at https://github.com/UF-IDD/US_County_Rt. Code for inference of parameters from the meta-population model is available upon request to cdlaird@sandia.gov.

## References

[1] GitHub - nytimes/covid-19-data: An ongoing repository of data on coronavirus cases and deaths in the U.S. https://github.com/nytimes/covid-19-data.

[2] Auger KA (2020). Association between Statewide School Closure and COVID-19 Incidence and Mortality in the US. JAMA.

[3] Miller IF, Becker AD, Grenfell BT, Metcalf CJE (2020). Disease and healthcare burden of COVID-19 in the United States. Nat. Med..

[4] Unwin, H. J. T. et al. State-level tracking of COVID-19 in the United States. *Nat. Commun*. **11**, 6189 (2020). 10.1038/s41467-020-19652-6 PMC7712910 33273462

[5] White, E. R. & Hébert-Dufresne, L. State-level variation of initial COVID-19 dynamics in the United States: The role of local government interventions. *medRxiv.*10.1101/2020.04.14.20065318 (2020). 10.1371/journal.pone.0240648 PMC7553297 33048967

[6] COVID-19 US State Policy Database. https://www.openicpsr.org/openicpsr/project/119446/version/V84/view;jsessionid=8AF894AA100673728A9D68E76DE1AC1D.

[7] COVID-19 Community Mobility Reports. https://www.google.com/covid19/mobility/.

[8] Flaxman, S. et al. Estimating the effects of non-pharmaceutical interventions on COVID-19 in Europe. *Nature.*10.1038/s41586-020-2405-7 (2020). 10.1038/s41586-020-2405-7 32512579

[9] Hsiang, S. et al. The effect of large-scale anti-contagion policies on the COVID-19 pandemic. *Nature.*10.1038/s41586-020-2404-8 (2020). 10.1038/s41586-020-2404-8 32512578

[10] Zhang J (2020). Changes in contact patterns shape the dynamics of the COVID-19 outbreak in China. Science.

[11] Brauner, J. M. et al. Inferring the effectiveness of government interventions against COVID-19. *Science*. eabd9338. 10.1126/science.abd9338 (2020). 10.1126/science.abd9338 PMC7877495 33323424

[12] Siedner MJ (2020). Social distancing to slow the US COVID-19 epidemic: Longitudinal pretest–posttest comparison group study. PLoS Med..

[13] Rader, B. et al. Mask-wearing and control of SARS-CoV-2 transmission in the USA: a cross-sectional study. *Lancet Digit. Heal*. **3**, e148–e157 (2021). 10.1016/S2589-7500(20)30293-4 PMC7817421 33483277

[14] Mitze T, Kosfeld R, Rode J, Walde K (2020). Face masks considerably reduce COVID-19 cases in Germany. Proc. Natl Acad. Sci. USA..

[15] Korevaar, H. M. et al. Quantifying the impact of US state non-pharmaceutical interventions on COVID-19 transmission. *medRxiv.*10.1101/2020.06.30.20142877 (2020).

[16] Liu Y (2021). The impact of non-pharmaceutical interventions on SARS-CoV-2 transmission across 130 countries and territories. BMC Med..

[17] Li Y (2021). The temporal association of introducing and lifting non-pharmaceutical interventions with the time-varying reproduction number (R) of SARS-CoV-2: a modelling study across 131 countries. Lancet Infect. Dis..

[18] Lessler, J. et al. Household COVID-19 risk and in-person schooling. *Science*. eabh2939. 10.1126/science.abh2939 (2021). 10.1126/science.abh2939 PMC8168618 33927057

[19] Pitzer, V. E. et al. The impact of changes in diagnostic testing practices on estimates of COVID-19 transmission in the United States. *medRxiv.*10.1101/2020.04.20.20073338 (2020). 10.1093/aje/kwab089 PMC8083380 33831148

[20] Ali ST (2020). Serial interval of SARS-CoV-2 was shortened over time by nonpharmaceutical interventions. Science.

[21] Yan Y (2021). Measuring voluntary and policy-induced social distancing behavior during the COVID-19 pandemic. Proc. Natl Acad. Sci..

[22] Fisher KA (2020). Community and Close Contact Exposures Associated with COVID-19 Among Symptomatic Adults ≥18 Years in 11 Outpatient Health Care Facilities—United States, July 2020. Mmwr. Morb. Mortal. Wkly. Rep..

[23] Jang S, Han SH, Rhee JY (2020). Cluster of Coronavirus disease associated with fitness dance classes, South Korea. Emerg. Infect. Dis..

[24] Walker, K., Eberwein, K. & Herman, M. Tidycensus: Load us census boundary and attribute data as’ tidyverse’and’sf’-ready data frames. *R package version 0.9*. 6. https://walker-data.com/tidycensus/ (2018).

[25] Becker NG, Watson LF, Carlin JB (1991). A method of non‐parametric back‐projection and its application to aids data. Stat. Med..

[26] Höhle M (2007). Surveillance: An R package for the monitoring of infectious diseases. Comput. Stat..

[27] Abbott S (2020). Estimating the time-varying reproduction number of SARS-CoV-2 using national and subnational case counts. Wellcome Open Res..

[28] Lemaitre, J. C. et al. A scenario modeling pipeline for COVID-19 emergency planning. *Sci. Rep*. **11**, 7534 (2021). 10.1038/s41598-021-86811-0 PMC8024322 33824358

[29] Census Bureau. Commuting Data—Census Bureau. https://www.census.gov/topics/employment/commuting.html.

[30] Hart, W. E. *et al*. *Pyomo—Optimization Modeling in Python*. (Springer Optimization and Its Applications, 2017).

[31] Wächter A, Biegler LT (2006). On the implementation of an interior-point filter line-search algorithm for large-scale nonlinear programming. Math. Program..

[32] Sanche, S. et al. The novel coronavirus, 2019-nCoV, is highly contagious and more infectious than initially estimated. *medRxiv.*10.1101/2020.02.07.20021154 (2020).

[33] Bi Q (2020). Epidemiology and transmission of COVID-19 in 391 cases and 1286 of their close contacts in Shenzhen, China: a retrospective cohort study. Lancet Infect. Dis..

[34] Anzai A (2020). Assessing the Impact of Reduced Travel on Exportation Dynamics of Novel Coronavirus Infection (COVID-19). J. Clin. Med..

[35] Verity R (2020). Estimates of the severity of coronavirus disease 2019: a model-based analysis. Lancet Infect. Dis..

[36] Halekoh U, Højsgaard S, Yan J (2006). The R package geepack for generalized estimating equations. J. Stat. Softw..

[37] CRAN—Package glmnet. https://cran.r-project.org/web/packages/glmnet/index.html.

[38] Chen, T., Guestrin, C. XGBoost: a scalable tree boosting system. in *Proceedings of the ACM SIGKDD International Conference on Knowledge Discovery and Data Mining*, Vols. 13–17. 785–794 (Association for Computing Machinery, 2016).

[39] Tingley, D., Yamamoto, T., Hirose, K., Keele, L. & Imai, K. mediation: R Package for Causal Mediation Analysis. *J. Stat. Softw*. 10.18637/jss.v059.i05 (2014).

