# Supplementary material for: Effect of specific non-pharmaceutical intervention policies on SARS-CoV-2 transmission in the counties of the United States
Source: Nat Commun. 2021 Jun 11;12:3560. doi: 10.1038/s41467-021-23865-8 (PMC8195990; doi:10.1038/s41467-021-23865-8)
Supplement: Supplementary file 1 — Supplementary Information [file 41467_2021_23865_MOESM1_ESM.pdf]

# Supplementary information

## Effect of specific non-pharmaceutical intervention policies on SARS-CoV-2 transmission in the counties of the United States

Bingyi Yang, Angkana T. Huang, Bernardo Garcia-Carreras, William E. Hart, Andrea Staid, Matt D.T. Hitchings, Elizabeth C. Lee, Chanelle J. Howe, Kyra H. Grantz, Amy Wesolowksi, Joseph Chadi Lemaitre, Susan Rattigan, Carlos Moreno, Brooke A. Borgert, Celeste Dale, Nicole Quigley, Andrew Cummings, Alizée McLorg, Kaelene LoMonaco, Sarah Schlossberg, Drew Barron-Kraus, Harrison Shrock, UFCOVID Interventions Team*, Justin Lessler, Carl D. Laird, Derek A.T. Cummings

**Correspondence to:**
Derek A.T. Cummings (datc@ufl.edu), Carl D. Laird (cdlaird@sandia.gov), Justin Lessler (justin@jhu.edu)

**Supplementary Tables**

**Supplementary Table 1.** Groupings of non-pharmaceutical interventions (NPIs) extracted from the COVID-19 US State Policy Database (CUSP).

| Interventions in dataset [#] | Groupings of interventions[†] |
|---|---|
| School closure | School closure |
| Daycare closure | Daycare closure |
| Nursing home visit ban | Nursing home visit ban |
| Face mask mandated in public<br>Face mask mandated in businesses | Face mask |
| Close restaurants<br>Close gyms<br>Close movie theaters | Leisure activities closure |
| Stay at home orders<br>Close non-essential businesses* | Stay at home |
| Suspend non-essential medical services | Suspend non-essential medical services |

[#] Data on non-pharmaceutical interventions (NPIs) directly targeting transmission reduction

[†] NPIs groupings used in all analyses; grouped based on correlated presence/absence and semantic similarity.

*Closure of non-essential businesses is grouped with stay at home orders because the effect of closing non-essential businesses is thought to prevent workers from visiting their workplace and most likely lead to them staying home. Closure of non-essential businesses was also highly correlated with stay at home orders (Supplementary Fig. 2).

**Supplementary Table 2.** Covariates included in the main model and sensitivity analyses.

| Covariates | | Whether or not included in the model | | | |
|---|---|---|---|---|---|
| **Category** | **Detailed covariate** | **Base** | **Main** | **Time only** | **Time and interventions** |
| **Autoregressive** | AR(1) for GEEs;<br>$Log_{10}$ $R_{eff-1}$ for OLS model | Yes | Yes | Yes | Yes |
| **County time** | Week -3 to 13 (categorical) | No | No | Yes | Yes |
| **County characteristics** | State-specific intercept<br>Median income<br>Median age<br>$Log_{10}$ population size<br>$Log_{10}$ population density<br>Decile of poverty<br>Decile of white<br>Decile of no college education | Yes | Yes | Yes | Yes |
| **NPIs** | School closure<br>Leisure activities closure<br>Stay at home<br>Daycare closure<br>Nursing home visit ban<br>Medical service suspension<br>Face mask | No | Yes | No | Yes |

**Supplementary Table 3.** Comparison of models with temporal markers and non-pharmaceutical interventions.

| Model | Covariates included[#] | | GEE[†] | | OLS[§] | |
|---|---|---|---|---|---|---|
| | Additional temporal marker | NPIs | Adj. $R^2$ | ΔQIC | Adj. $R^2$ | ΔAIC |
| **Base** | -- | No | 7.8% | Ref | 25.0% | Ref |
| **Main** | -- | Yes | 22.1% | -411.9 | 29.0% | -1681.9 |
| **Time only** | Weeks since county's first case (categorical) | No | 19.0% | -301.1 | 26.3% | -537.0 |
| **Time and interventions** | Weeks since county's first case (categorical) | Yes | 24.3% | -414.9 | 29.5% | -1908.2 |

[#] All models include county-level census information (i.e., population size, median income and decimal of poverty).

[†] Autocorrelation of $R_{eff}$ was adjusted using a correlation structure of AR(1) in the models.

[§] Autocorrelation of $R_{eff}$ was adjusted by including prior week's $R_{eff}$ as a covariate in the models.

**Supplementary Table 4.** Prediction performance evaluated through out-of-sample validation compared against a comparator high-complexity model given equivalent sets of covariates.

| Model type | Median performance (95% IQR) | | |
|---|---|---|---|
| | RMSE | MASE | $R^2$ |
| Main model (OLS model) | 0.31 (0.27, 0.36) | 0.83 (0.81, 0.86) | 0.21 (0.15, 0.29) |
| Comparator model (XGBoost) | 0.33 (0.13, 0.44) | 0.84 (0.75, 0.97) | 0.22 (0.03, 0.75) |

* One of the fifty US states or District of Columbia was held out from the training set each time. Performances were computed from the held-out data, i.e., the test set. Metrics measured are root mean squared error (RMSE), mean absolute scaled error (MASE) and coefficient of determination ($R^2$). Values are given as the median across the 51 spatial units with 95% interquartile in parentheses.

**Supplementary Table 5.** Associations between non-pharmaceutical interventions (NPIs) and county-level characteristics on transmission. Estimates of the proportional reduction associated with each NPI or county-level characteristic for both GEEs and OLS models.

| Variable | GEEs | OLS |
|---|---|---|
| School closure | 0.37 (0.33, 0.40) | 0.23 (0.20, 0.27) |
| Leisure activities closure | 0.14 (0.11, 0.16) | 0.14 (0.12, 0.16) |
| Stay at home | 0.15 (0.13, 0.17) | 0.09 (0.07, 0.11) |
| Face mask | 0.18 (0.16, 0.20) | 0.09 (0.07, 0.11) |
| Daycare closure | 0.31 (0.26, 0.35) | 0.23 (0.19, 0.27) |
| Nursing home visit ban | 0.26 (0.23, 0.29) | 0.12 (0.09, 0.16) |
| Medical service suspension | 0.00 (-0.03, 0.03) | 0.02 (-0.00, 0.04) |
| $Log_{10}$ population size | -0.16 (-0.24, -0.09) | -0.14 (-0.18, -0.11) |
| Decile of white | 0.02 (0.01, 0.02) | 0.01 (0.01, 0.02) |
| Median age | 0.01 (0.01, 0.01) | 0.01 (0.00, 0.01) |
| Decile of below college | -0.05 (-0.06, -0.04) | -0.04 (-0.05, -0.03) |
| Decile of poverty | -0.01 (-0.04, 0.02) | -0.01 (-0.03, 0.02) |
| Log population density | -0.02 (-0.08, 0.02) | -0.02 (-0.05, 0.01) |
| Median income | 0.07 (-0.05, 0.17) | 0.02 (-0.09, 0.12) |

Note: Reductions were calculated as $1 - 10^{\beta}$,where $\beta$ is the estimated coefficient for individual NPI.

**Supplementary Table 6.** Coefficients estimated in Figure 4.

| Suite | P, % | Interventions* | | | | | | | Estimates $R_{eff}$ (median, 95% quantiles) | | |
|---|---|---|---|---|---|---|---|---|---|---|---|
| | | DC | FM | LA | SH | NH | MS | SC | GEE individual | GEE suite | XGBoost |
| 1 | 8.5% | 0 | 0 | 0 | 0 | 0 | 0 | 0 | 2.70 (1.65, 4.52) | 6.47 (3.91, 9.31) | 4.53 (2.23, 7.26) |
| 2 | 6.5% | 0 | 0 | 0 | 0 | 0 | 0 | 1 | 1.72 (1.05, 2.87) | 2.08 (1.26, 3.00) | 1.17 (0.55, 5.19) |
| 3 | 4.2% | 0 | 0 | 0 | 0 | 1 | 0 | 1 | 1.27 (0.77, 2.12) | 1.41 (0.85, 2.03) | 1.04 (0.49, 5.18) |
| 4 | 8.7% | 0 | 1 | 0 | 0 | 1 | 0 | 1 | 1.04 (0.63, 1.74) | 1.11 (0.67, 1.60) | 1.05 (0.50, 4.91) |
| 5 | 6.3% | 0 | 0 | 1 | 0 | 1 | 0 | 1 | 1.09 (0.67, 1.83) | 1.14 (0.69, 1.64) | 0.97 (0.44, 4.89) |
| 6 | 4.7% | 0 | 0 | 1 | 1 | 0 | 0 | 1 | 1.26 (0.77, 2.11) | 1.43 (0.86, 2.06) | 0.91 (0.39, 4.71) |
| 7 | 6.7% | 0 | 0 | 1 | 1 | 1 | 0 | 1 | 0.93 (0.57, 1.56) | 0.97 (0.58, 1.39) | 0.89 (0.39, 4.72) |
| 8 | 4.3% | 0 | 0 | 1 | 1 | 0 | 1 | 1 | 1.26 (0.77, 2.11) | 1.42 (0.85, 2.04) | 0.89 (0.37, 4.93) |
| 9 | 4.2% | 0 | 1 | 1 | 0 | 1 | 0 | 1 | 0.90 (0.55, 1.50) | 0.97 (0.58, 1.39) | 0.97 (0.45, 5.01) |
| 10 | 4.5% | 1 | 0 | 1 | 1 | 1 | 1 | 1 | 0.65 (0.39, 1.08) | 0.78 (0.47, 1.13) | 0.85 (0.36, 4.95) |

* DC, daycare closure; FM, face mask; LA, leisure activities closure; SH, stay home; NH, nursing home visit ban; MS, medical service suspension; SC, school closure.

**Supplementary Table 7.** Effects of confirmation rate on the estimated effective reproduction numbers.

| Assumptions | % in the range* | Spearman correlation^ |
|---|---|---|
| Estimated from cases | | |
| A lower constant reporting probability (1/8) | 95.4% | 0.810 |
| A higher constant reporting probability (1/12) | 96.4% | 0.818 |
| Time-varying reporting probability | 95.5% | 0.794 |
| Estimated from deaths | | |
| A lower constant reporting probability (1/240) | 90.2% | 0.795 |
| A higher constant reporting probability (1/160) | 93.0% | 0.794 |
| Time-varying reporting probability | 92.3% | 0.789 |

* The percentage of $R_{eff}$ estimated from the new assumption is in the range of $R_{eff}$ estimated in the main analysis (for cases) or Supplementary Fig. 23 (for deaths) across 100 simulations for each county in each week.

^ Two-sided P-value.

**Supplementary Table 8.** Univariable analysis on county-level characteristics and weekly $R_{eff}$.

| | **Relative changes in $R_{eff}$, % (95% confidence interval)** | **Adjusted $R^2$** |
|---|---|---|
| Median household income | -4.3 (-1.4, 10.4) | 0.00% |
| Decile of poverty | 4.7 (3.3, 6.2) | 0.11% |
| $Log_{10}$ population density | 7.1 (5.8, 8.4) | 0.32% |
| $Log_{10}$ population size | 10.6 (9.1, 12.2) | 0.54% |
| Median age | -0.9 (-1.1, -0.7) | 0.27% |
| Decile of percentage of population that is white | -3.9 (-4.4, -3.4) | 0.63% |
| Decile of percentage of population for which highest educational attainment is high school | -1.8 (-2.6, -1.0) | 0.05% |

## Supplementary Figures

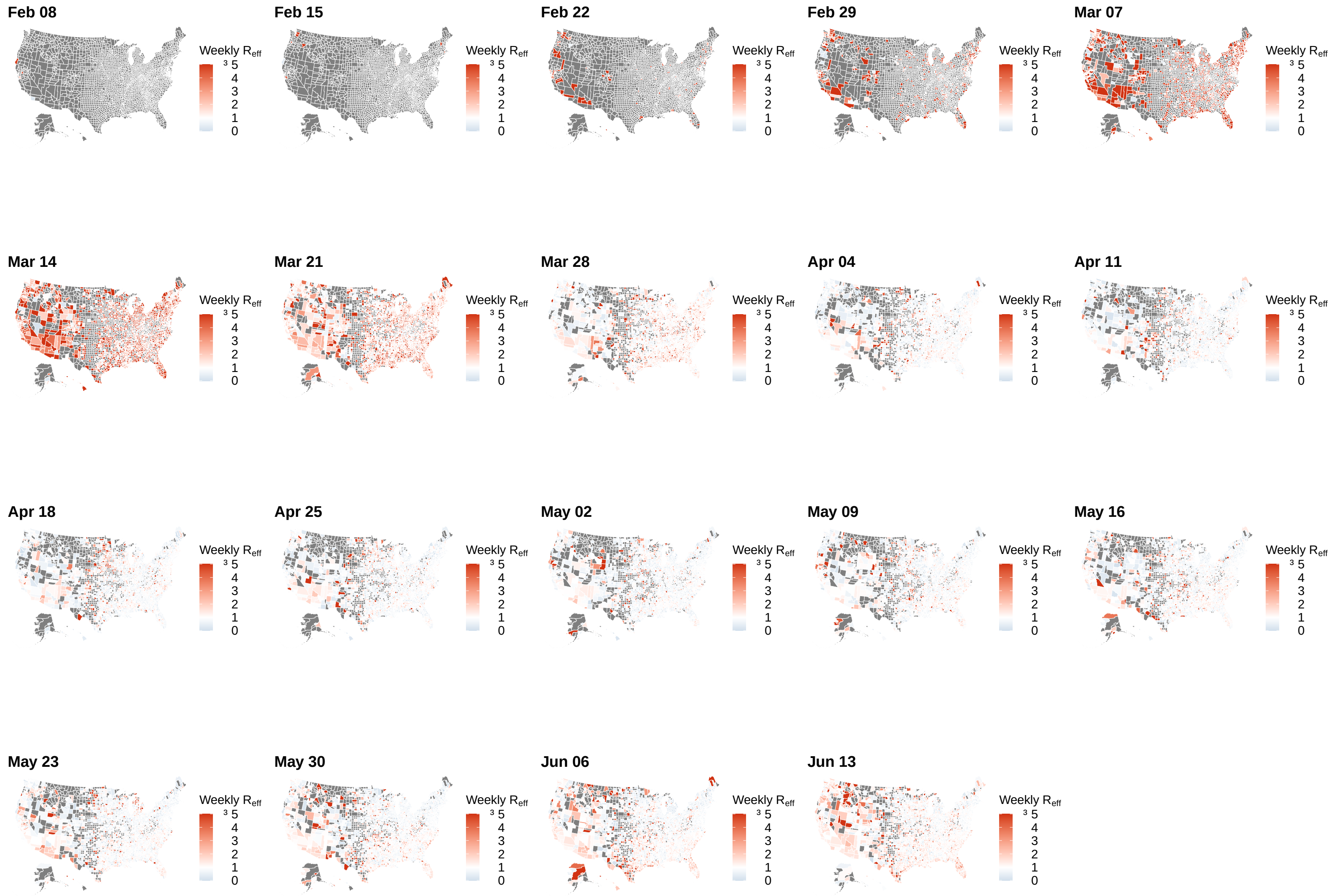


**Supplementary Figure 1. Maps of weekly reproduction number.** Grey indicates no data available.

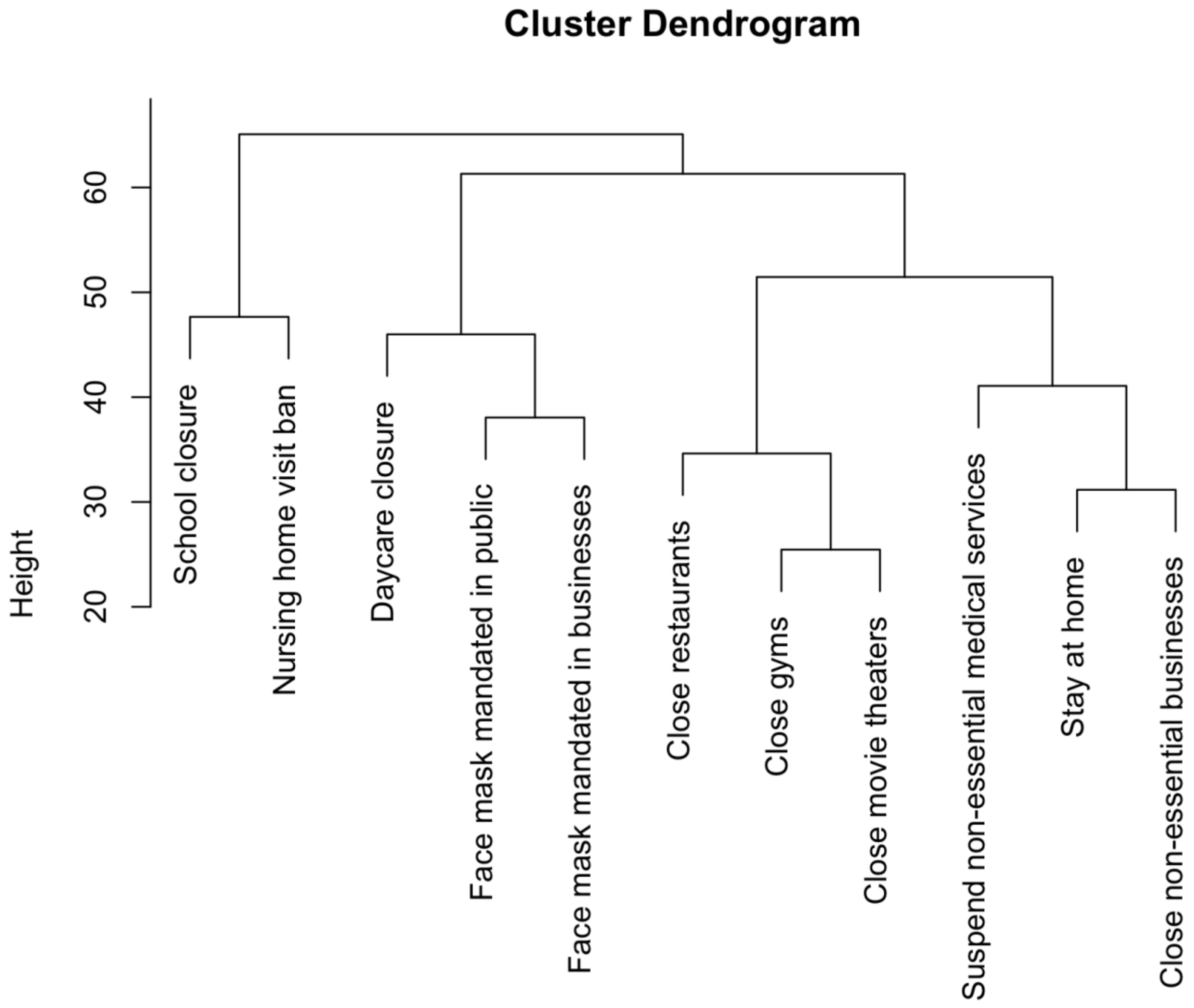


**Supplementary Figure 2. Hierarchical clustering of non-pharmaceutical interventions (NPIs) occurrences in time.**

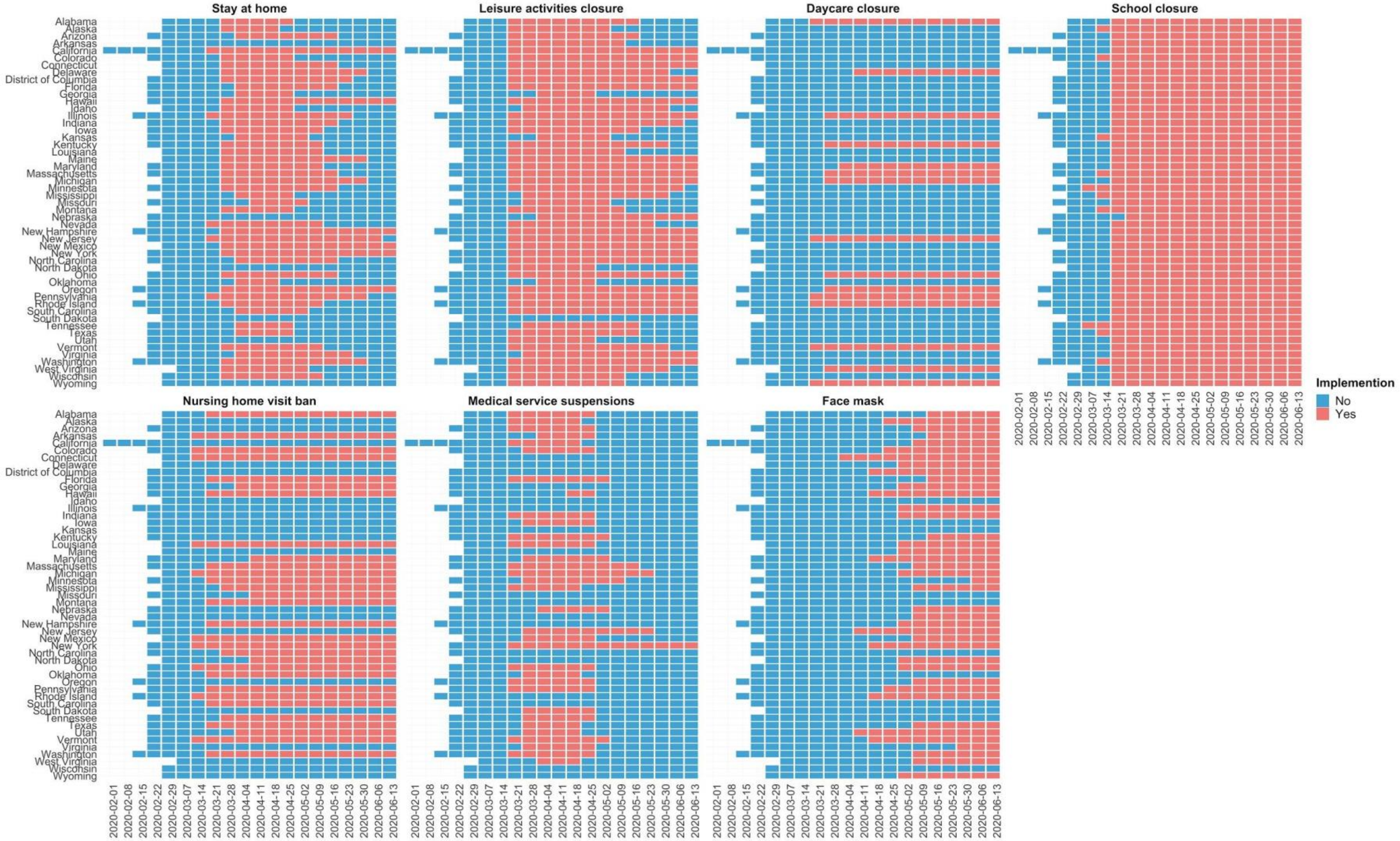


**Supplementary Figure 3. Temporal distribution of intervention by state.** Shown for weeks when $R_{eff}$ estimates were available.

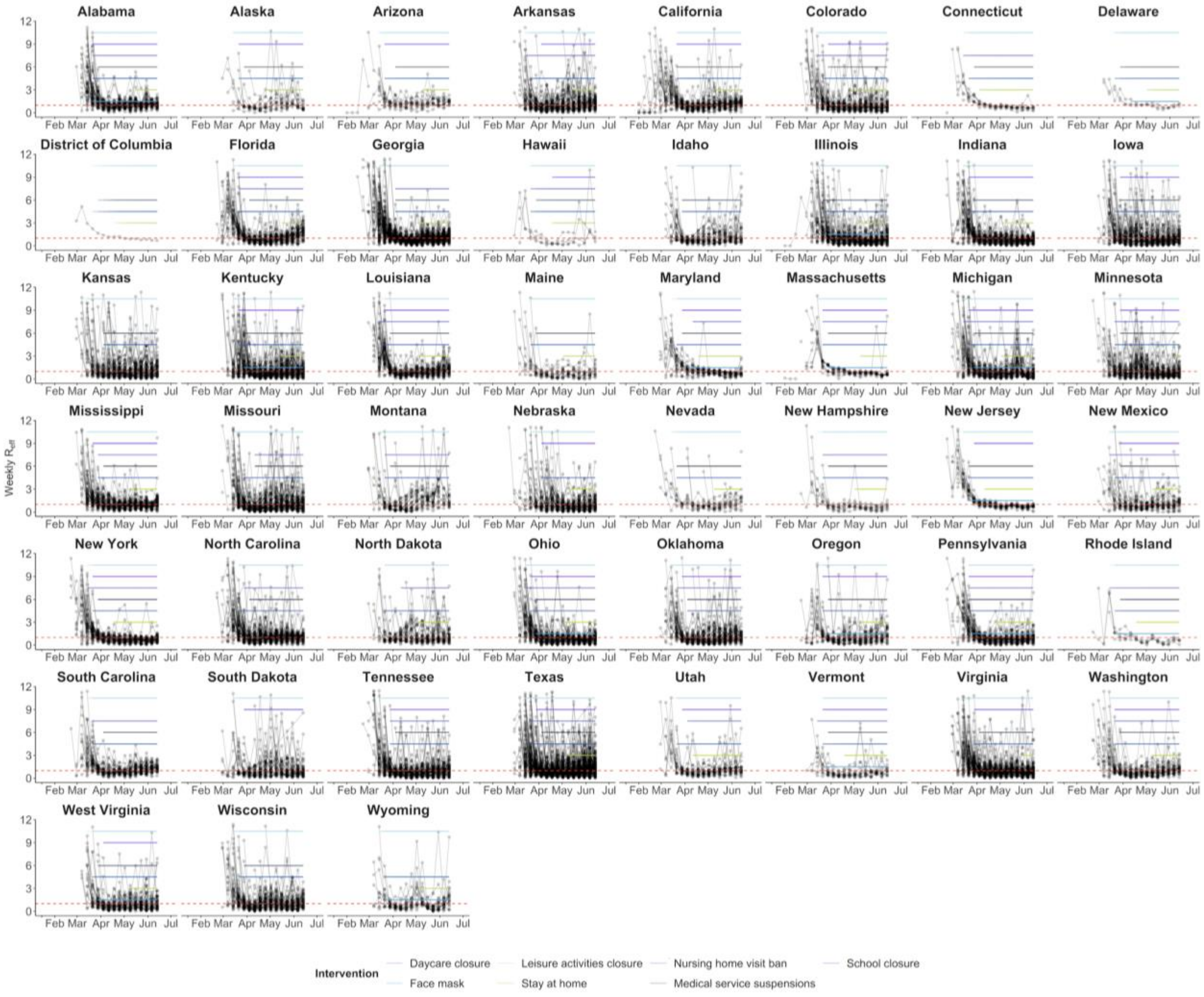


**Supplementary Figure 4: Estimates of $R_{eff}$ estimates from confirmed cases from each county organized by state.** County-specific time series of the median $R_{eff}$ across 100 estimations are shown. Colored solid lines indicate the duration of interventions implementation in each state. Red dashed line indicates a reproductive number of one.

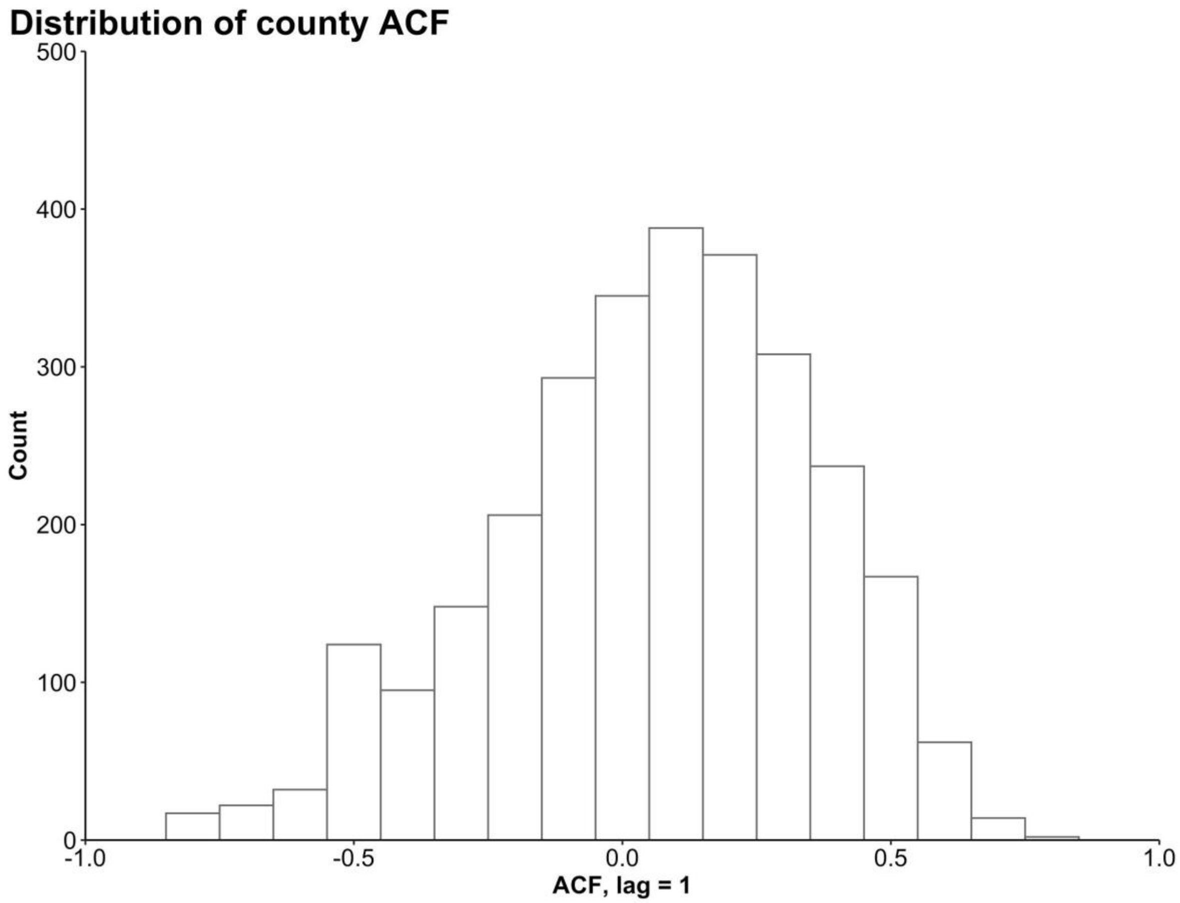


**Supplementary Figure 5. Distribution of county-specific autocorrelations of residuals of effective reproduction.** $R_{eff}$ were estimated from the main ordinary least squares (OLS) model but removed the $\log_{10} R_{eff}$ in the previous week.

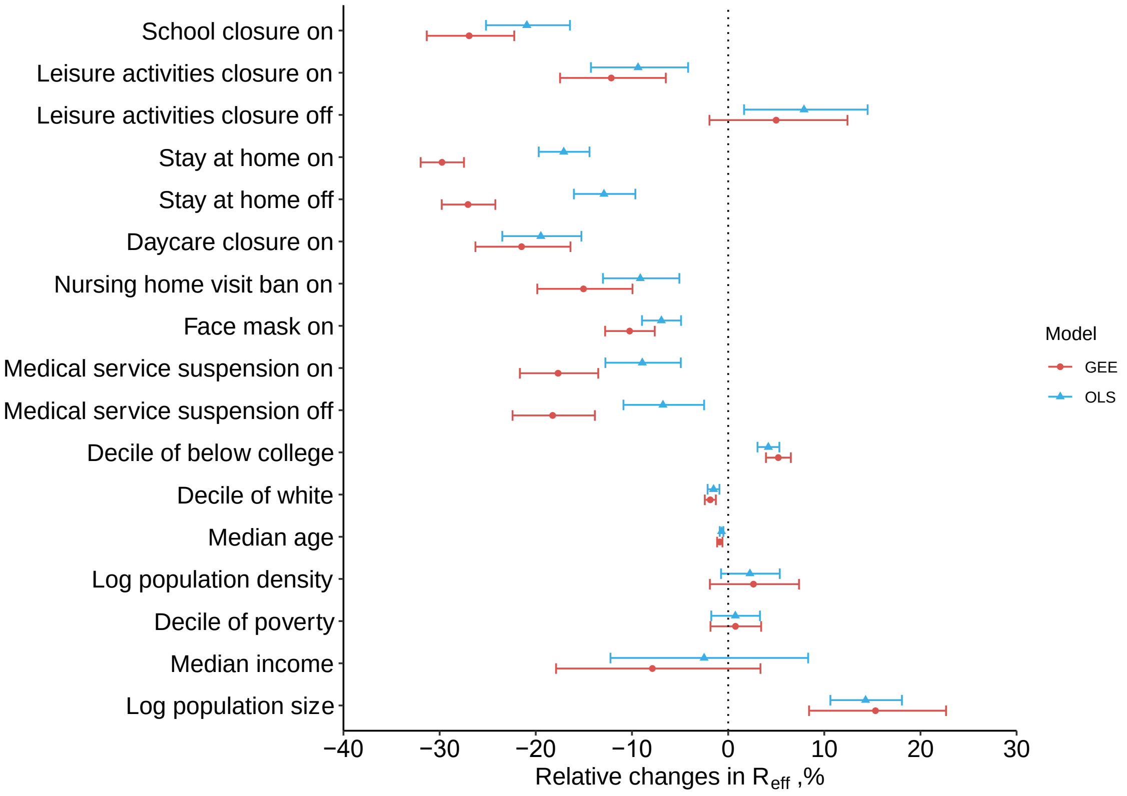


**Supplementary Figure 6. Associations between relaxing non-pharmaceutical interventions (NPIs) on transmission.** Main models were fitted by adding additional covariates to indicate the relaxation of NPIs when applicable (i.e., leisure activities closure, stay at home order and medical service suspension) (n = 31,072 county-weeks). Estimates from generalized estimation equations (GEEs) and ordinary least squares (OLS) were shown in red and blue, respectively. Data are presented as mean and 95% confidence interval.

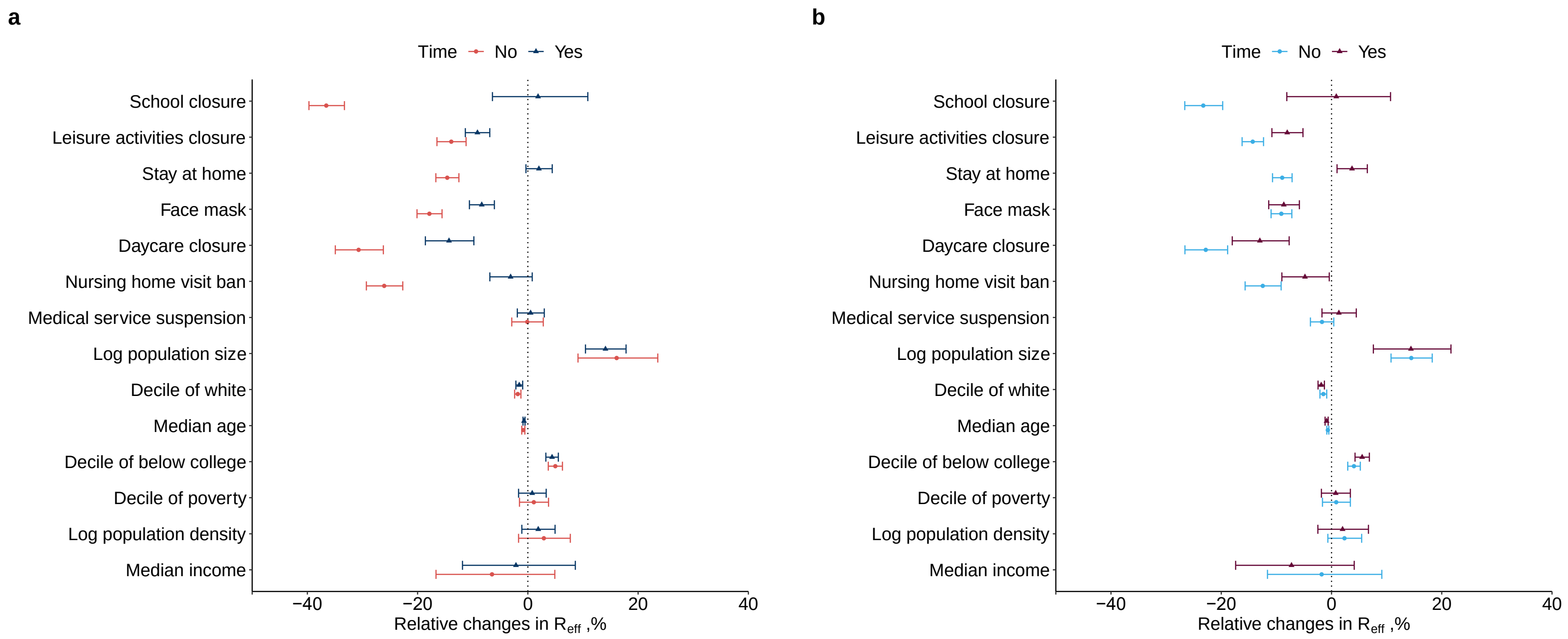


**Supplementary Figure 7. Associations between non-pharmaceutical interventions (NPIs) estimated from generalized estimating equations (GEEs) (a) and ordinary least squares (OLS) model (b) with and without calendar time.** Calendar time was modelled as categorical variables, which indicate the number of weeks since the United States saw its first case (n = 31,072 county-weeks). The shown models without calendar time are main models fitted with GEEs (a) and OLS models (details in Table S2). Data are presented as mean and 95% confidence interval.

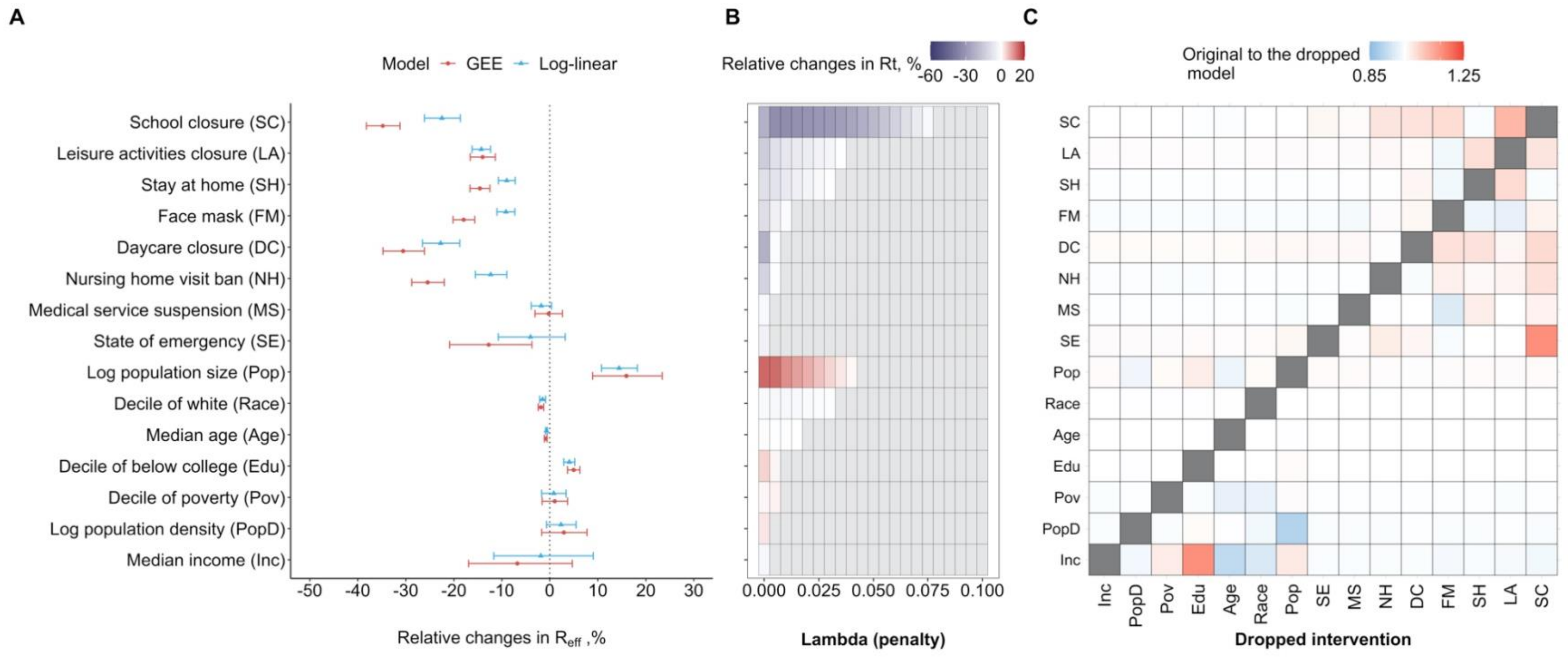


**Supplementary Figure 8. Associations between non-pharmaceutical interventions (NPIs) and county-level characteristics on transmission, including state of emergency. a**) Associations between NPIs and county-level characteristics estimated from the main model. Models were fitted with both generalized estimation equations (GEEs) and ordinary least squares (OLS) models (n = 31,072 county-weeks). Data are presented as mean and 95% confidence interval. The order in y-axis (same for c) is according to the importance of covariates in explaining the variances shown in b. **b**) The importance of covariates in explaining the variances. Main models that were formulated for OLS models and fitted to least absolute shrinkage and selection operator (LASSO) with increasing parsimony. **c**) Changes in the estimated effects when each covariate is dropped in the main OLS model.

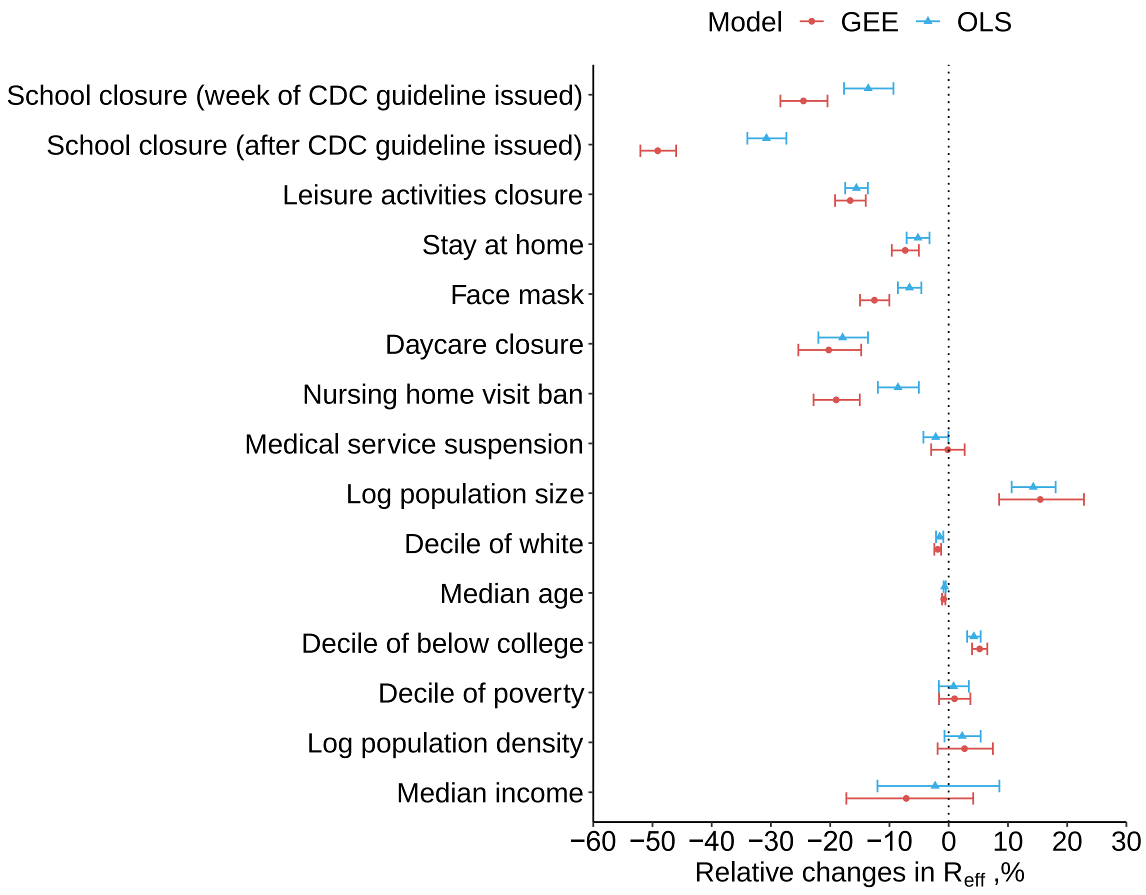


**Supplementary Figure 9. Possible effects of the overlapped time when CDC issued guidelines and school closure on the association between non-pharmaceutical interventions (NPIs) and SARS-CoV-2 transmissions.** CDC first issued their guidelines on 16 March 2020, while 83% of counties in our analysis closed schools in the same week (n = 31,072 county-weeks). Data are presented as mean and 95% confidence interval.

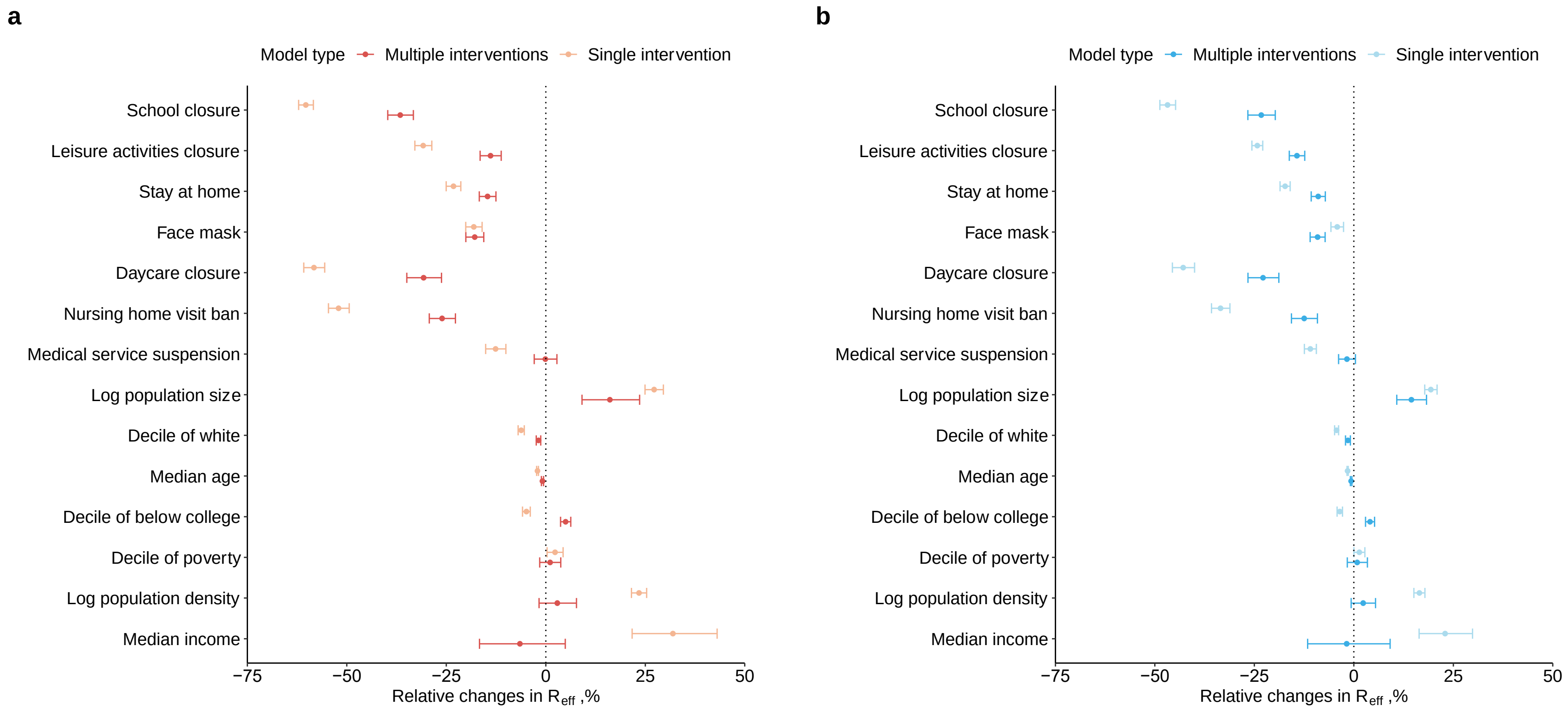


**Supplementary Figure 10. Comparison of associations between non-pharmaceutical interventions (NPIs) on transmission that were estimated from single-intervention (univariate) and multi-intervention models (multivariate).** For single-intervention models, we added one intervention to the base model (Supplementary Table 2) at a time. Models were fitted with generalized estimation equations (GEEs, a) and ordinary least squares (OLS, b) model (n = 31,072 county-weeks). Data are presented as mean and 95% confidence interval.

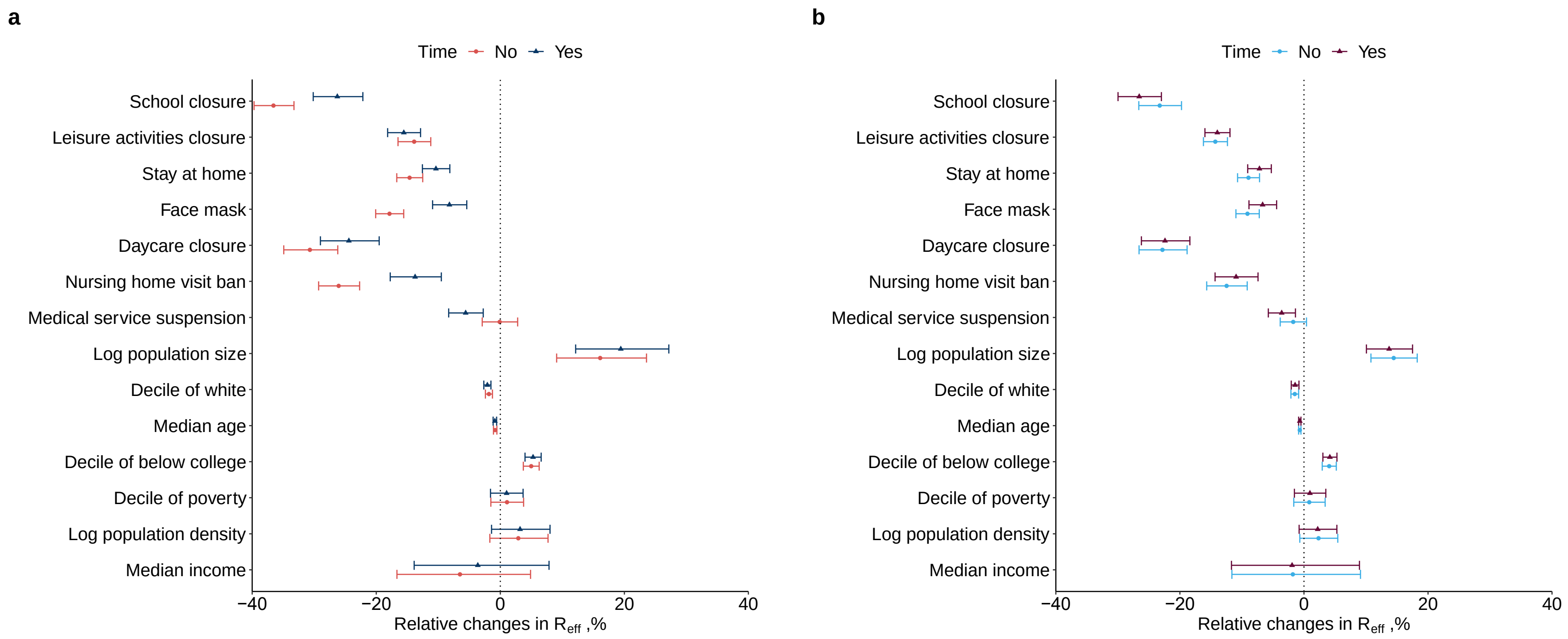


**Supplementary Figure 11. Associations between non-pharmaceutical interventions (NPIs) estimated from generalized estimating equations (GEEs) (a) and ordinary least squares (OLS) model (b) with and without county-specific time, proxied by the county saw its first case.** County-specific time was modelled as categorical variables, which indicate the number of weeks since the county saw its first case (Supplementary Table 2; see Methods). The shown models without county-specific time are main models fitted with GEEs (a) and OLS model(b) (n = 31,072 county-weeks; details in Supplementary Table 2). Data are presented as mean and 95% confidence intervals.

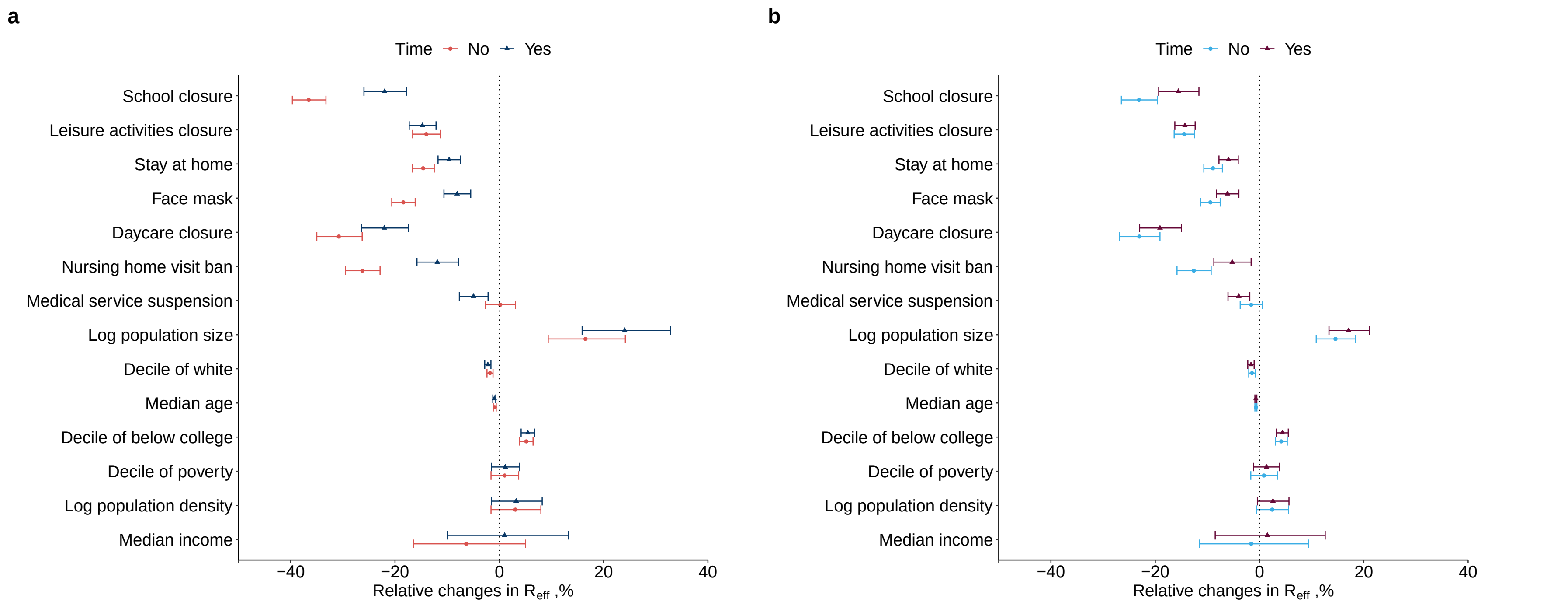


**Supplementary Figure 12. Associations between non-pharmaceutical interventions (NPIs) estimated from generalized estimating equations (GEEs) and ordinary least squares (OLS) model with and without county-specific time, proxied by the county saw its first 10 cases.** County-specific time was modelled as categorical variables, which indicate the number of weeks since the county saw its first case (Supplementary Table 2; see Methods). The shown models without county-specific time are main models fitted with GEEs (a) and OLS model (b) (n = 31,072 county-weeks; details in Supplementary Table 2). Data are presented as mean and 95% confidence interval.

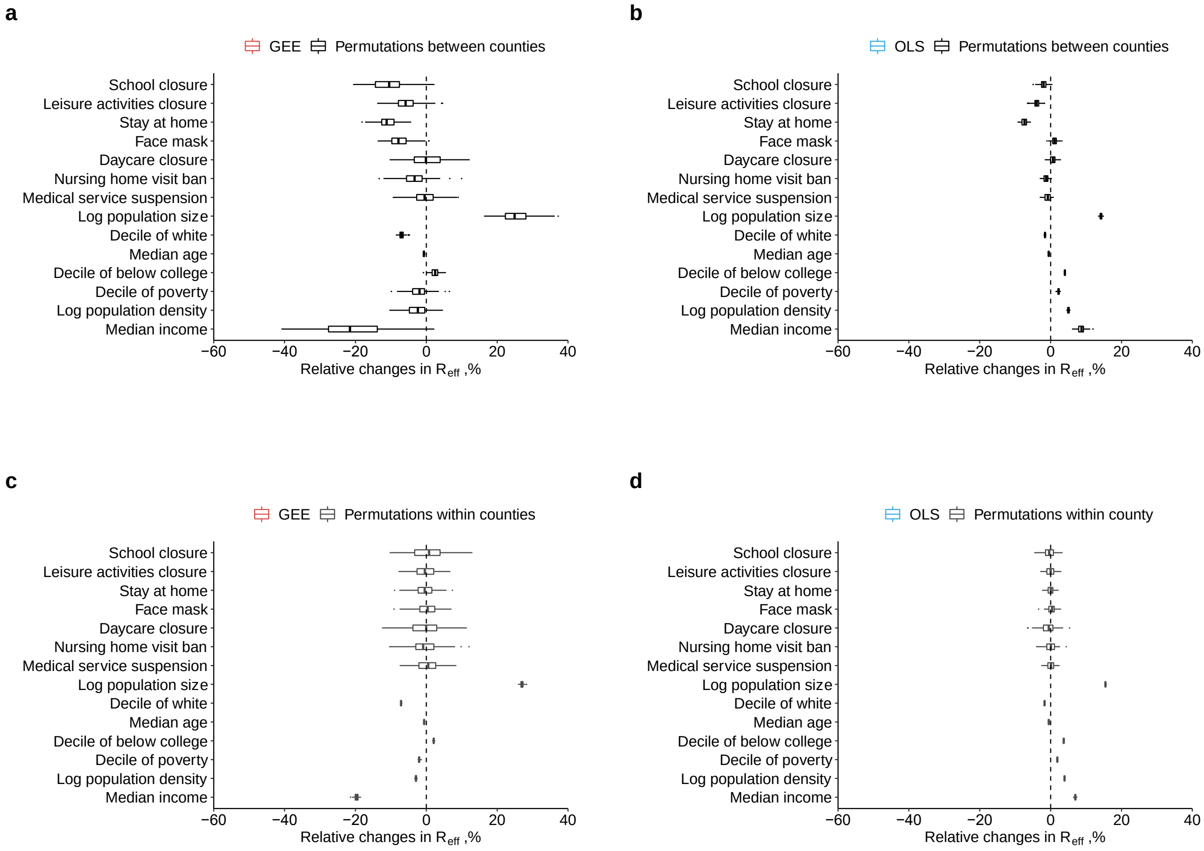


**Supplementary Figure 13. Associations between non-pharmaceutical interventions (NPIs) estimated from the main models compared against estimates when county-level NPIs suites were permuted spatially (a, b for GEEs and OLS model, respectively) or temporally (c, d for GEEs and OLS model respectively).** GEEs: generalized estimation equations. OLS: ordinary least squares. Colored estimates are presented as mean and 95% confidence interval of estimates from 31,072 county-weeks. Box plots represent median, interquartile and 95%intervals from 100 permutations.

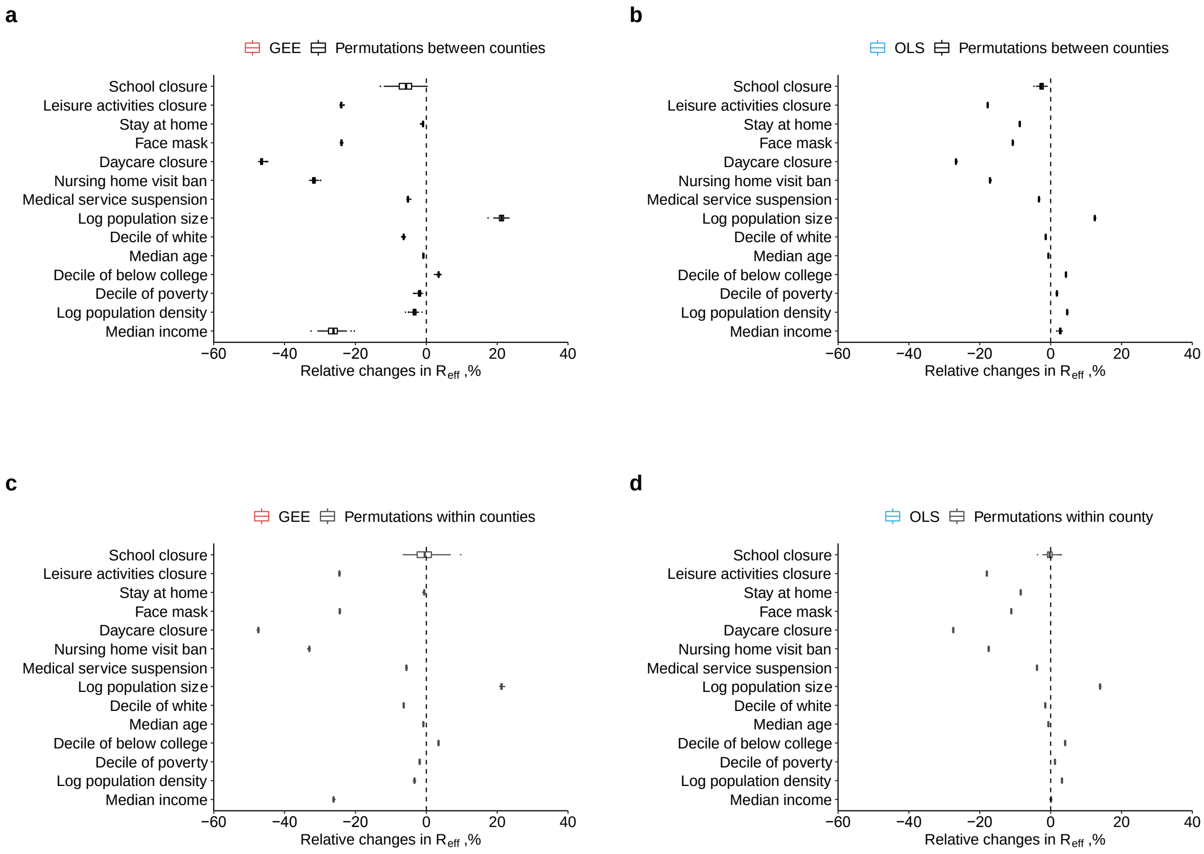


**Supplementary Figure 14. Associations between non-pharmaceutical interventions (NPIs) estimated from the main models compared against estimates when county-level school closures were permuted spatially (a, b for GEE and OLS model, respectively) or temporally (c, d for GEE and OLS model respectively).** GEEs: generalized estimation equations. OLS: ordinary least squares. Colored estimates are presented as mean and 95% confidence interval of estimates from 31,072 county-weeks. Box plots represent median, interquartile and 95% intervals from 100 permutations.

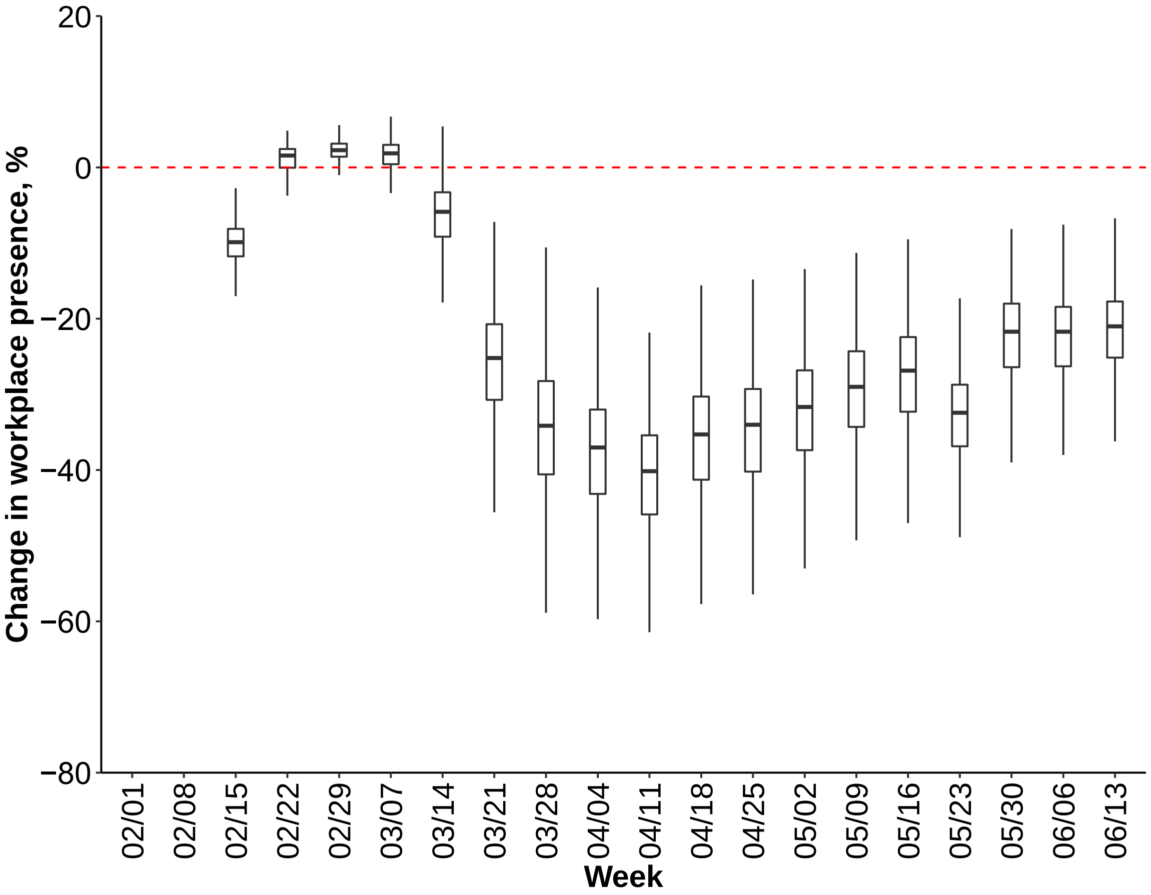


**Supplementary Figure 15. Temporal distribution of changes in workplace presence from Google data.** Data on workplace presence relative to pre-pandemic periods [7]. Data are presented as median (thick line), interquartile (IQR, box) and 1.5 times of the IQR for 36,737 county-weeks.

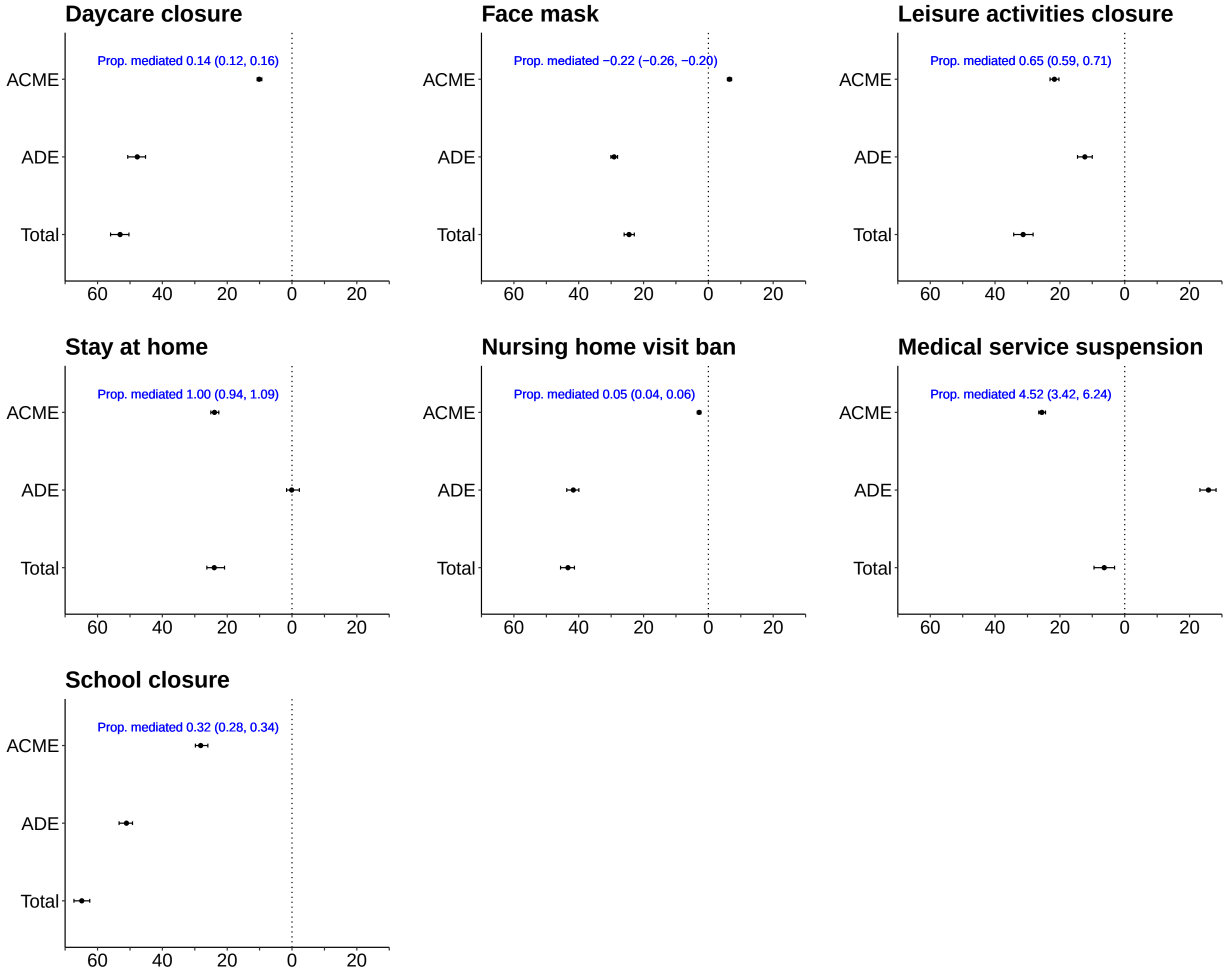


**Supplementary Figure 16. Mediation analyses of workplace presence on the association between non-pharmaceutical interventions (NPIs) and SARS-CoV-2 transmissions.** Models were fitted in ordinary least squares (OLS) model and adjusted for county-level characteristics and autocorrelation of $\log_{10} R_{eff}$ (n = 31,072 county-weeks). Data are presented as mean and 95% confidence interval. ACME, average causal mediation effect; ADE, average direct effect; Total, total effect.

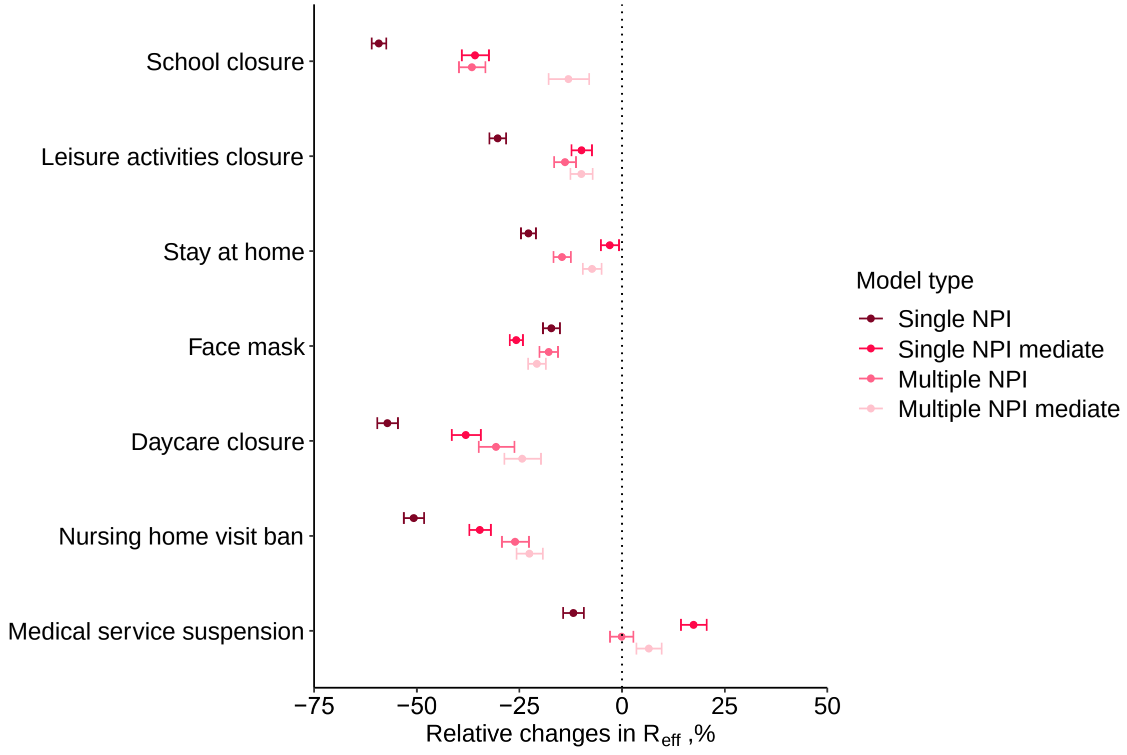


**Supplementary Figure 17. Effects of workplace presence on the association between non-pharmaceutical interventions (NPIs) and SARS-CoV-2 transmissions.** All models were fitted with generalized estimation equations (GEEs) and adjusted for county-level characteristics and autocorrelation of $\log_{10} R_{eff}$ (n = 31,072 county-weeks). Data are presented as mean and 95% confidence interval. Single NPI model includes the examined NPI; single NPI mediate model includes the examined NPI and the workplace presence; multiple NPI mediate model includes all NPIs and the workplace presence.

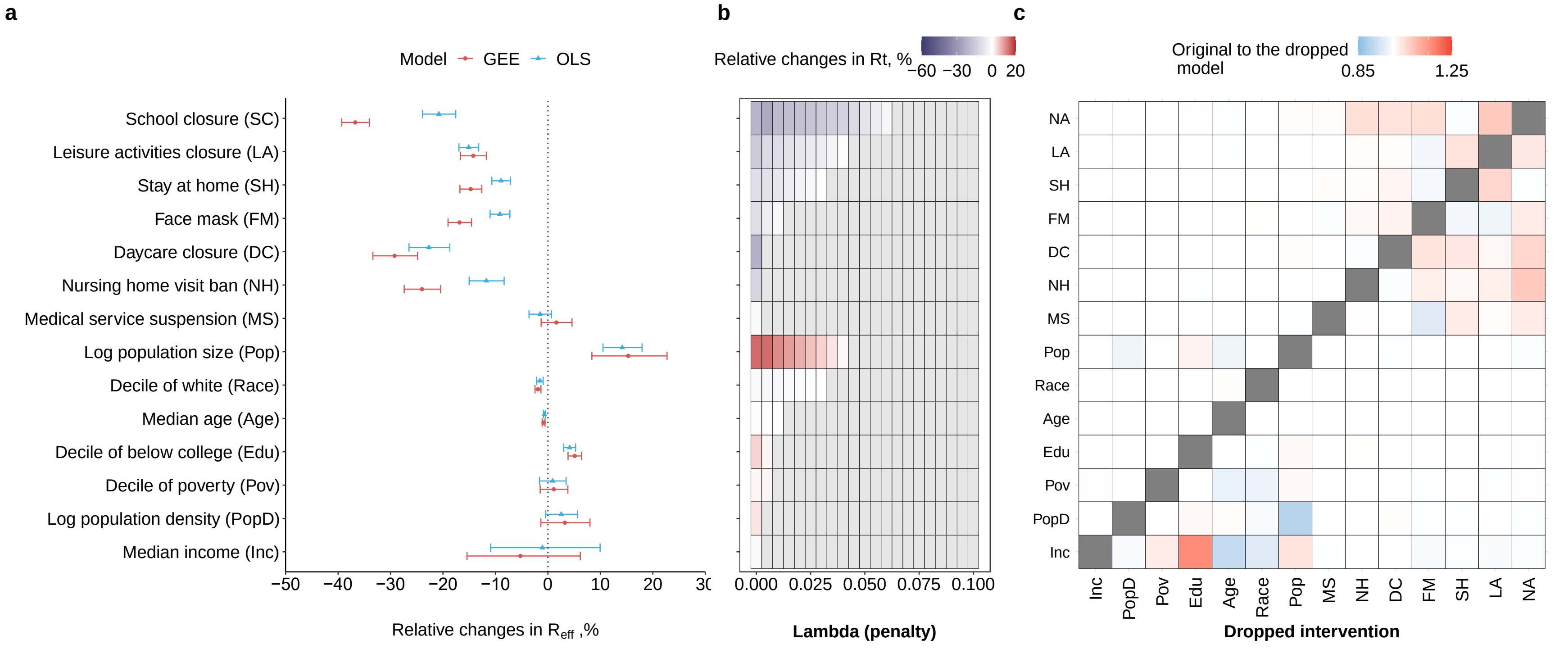


**Supplementary Figure 18. Associations between non-pharmaceutical interventions (NPIs) and county-level characteristics on transmission, using state-level school closure. a**) Effects of NPIs and county-level characteristics estimated from the main model (n = 31,072 county-weeks). Models were fitted with both generalized estimation equations (GEEs) and ordinary least squares (OLS) models. Data are presented as mean and 95% confidence interval. The order in y-axis (same for c) is according to the importance of covariates in explaining the variances shown in b. **b**) The importance of covariates in explaining the variances. Main models that were formulated for OLS models and fitted to least absolute shrinkage and selection operator (LASSO) with increasing parsimony. **c**) Changes in the estimated effects when each covariate is dropped in the main OLS model.

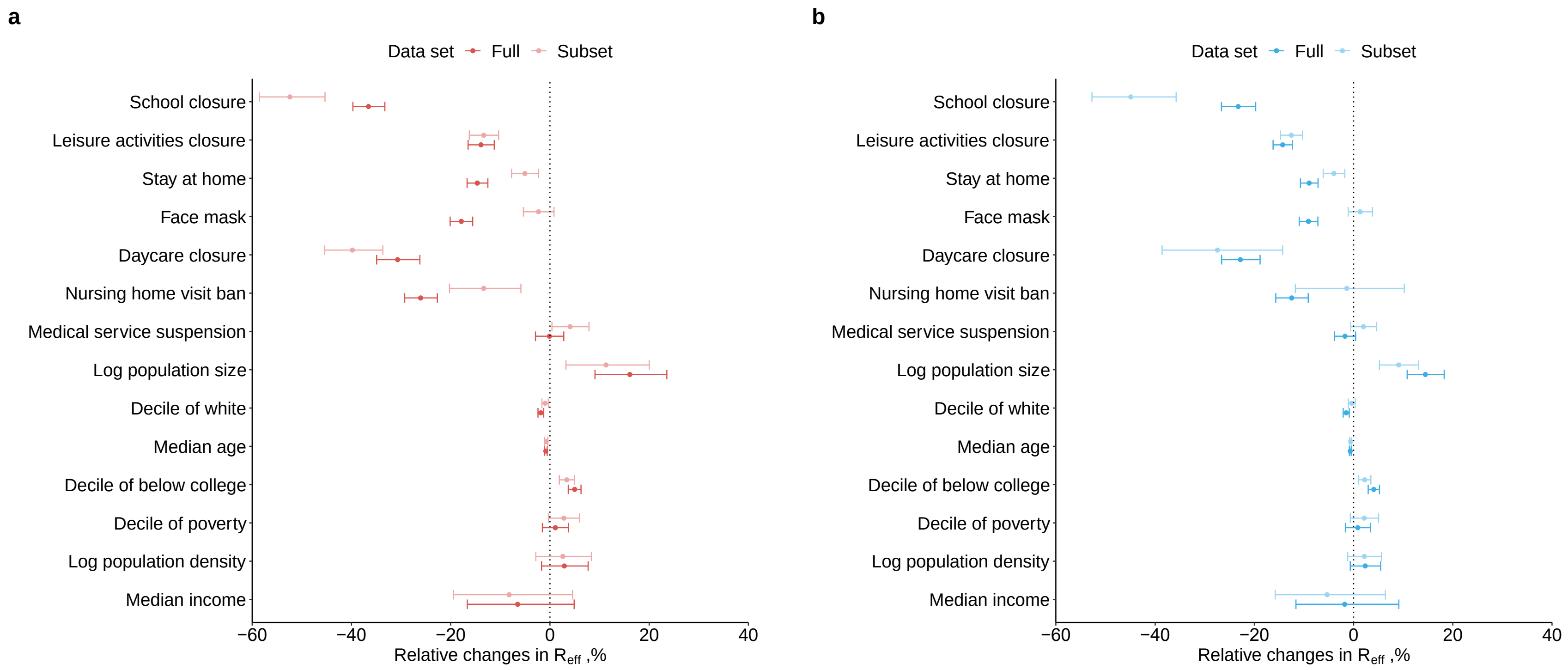


**Supplementary Figure 19. Associations between non-pharmaceutical interventions (NPIs) estimated with effective reproduction number ($R_{eff}$) estimates subsetted to two weeks after the county's first case onwards (n = 23,209 county-weeks) compared to the full dataset (n = 31,072 county-weeks) from generalized estimating equations (GEEs, a) and ordinary least squares (OLS, b) model.** Data are presented as mean and 95% confidence interval.

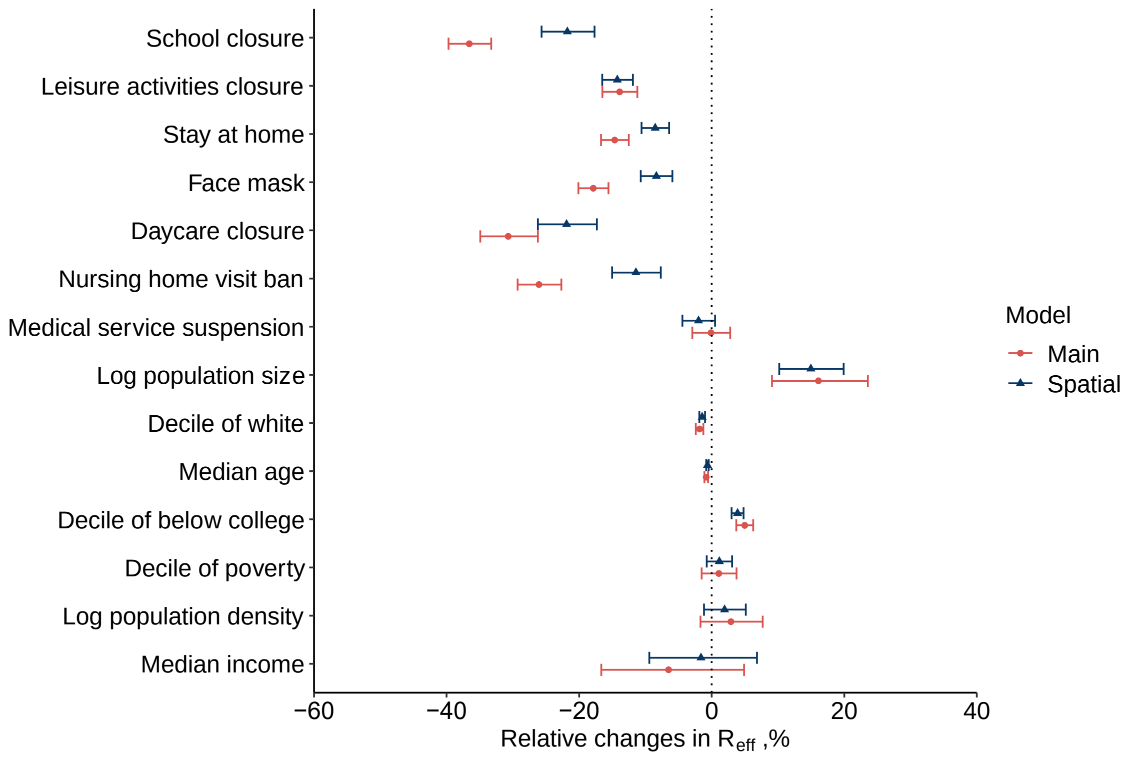


**Supplementary Figure 20. Effects of spatial correlation on the association between non-pharmaceutical interventions (NPIs) and SARS-CoV-2 transmissions.** Model was fitted with generalized estimation equations (GEEs) and adjusted for county-level characteristics, autocorrelation of $\log_{10} R_{eff}$ and spatial clustering (details in methods). Data are presented as mean and 95% confidence interval for estimates from 31,072 county-weeks.

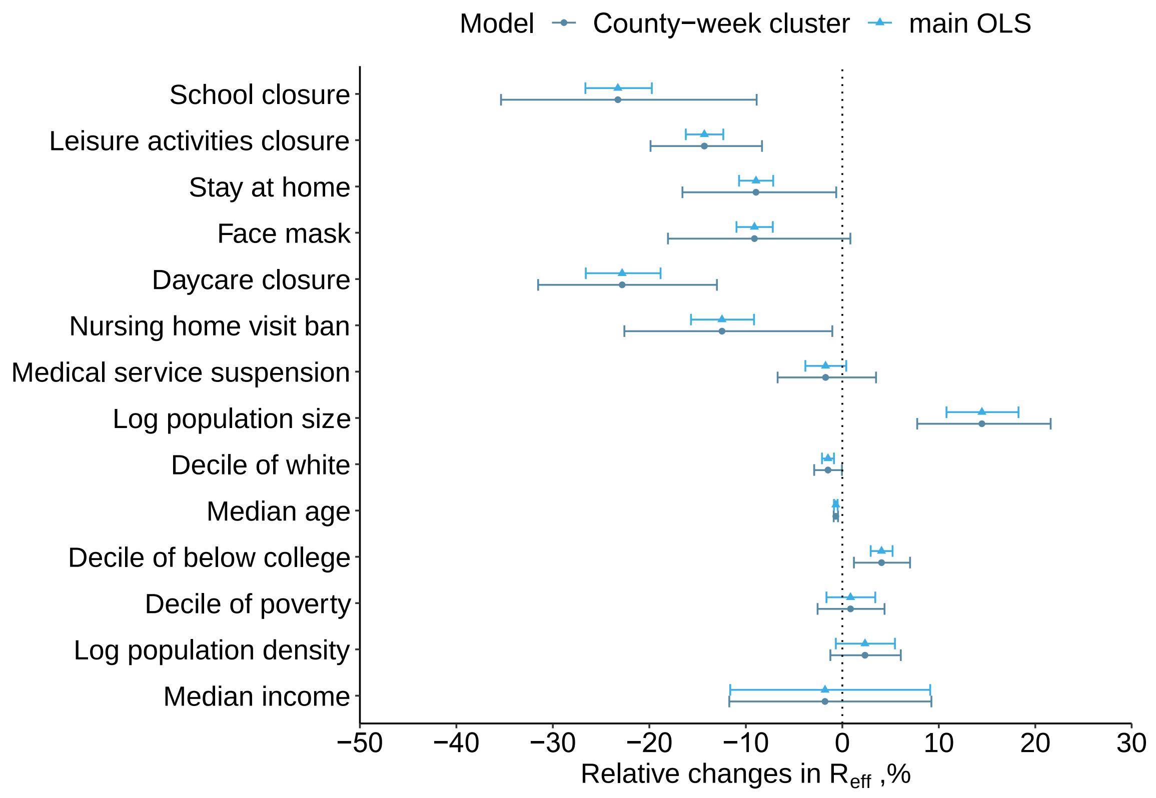


**Supplementary Figure 21. Effects of spatial-temporal clustering on the association between non-pharmaceutical interventions (NPIs) and SARS-CoV-2 transmissions.** We refitted the main ordinary least squares (OLS) model and calculated for cluster-robust standard errors (i.e., two-way clustered of county and time). Data are presented as mean and 95% confidence interval for estimates from 31,072 county-weeks.

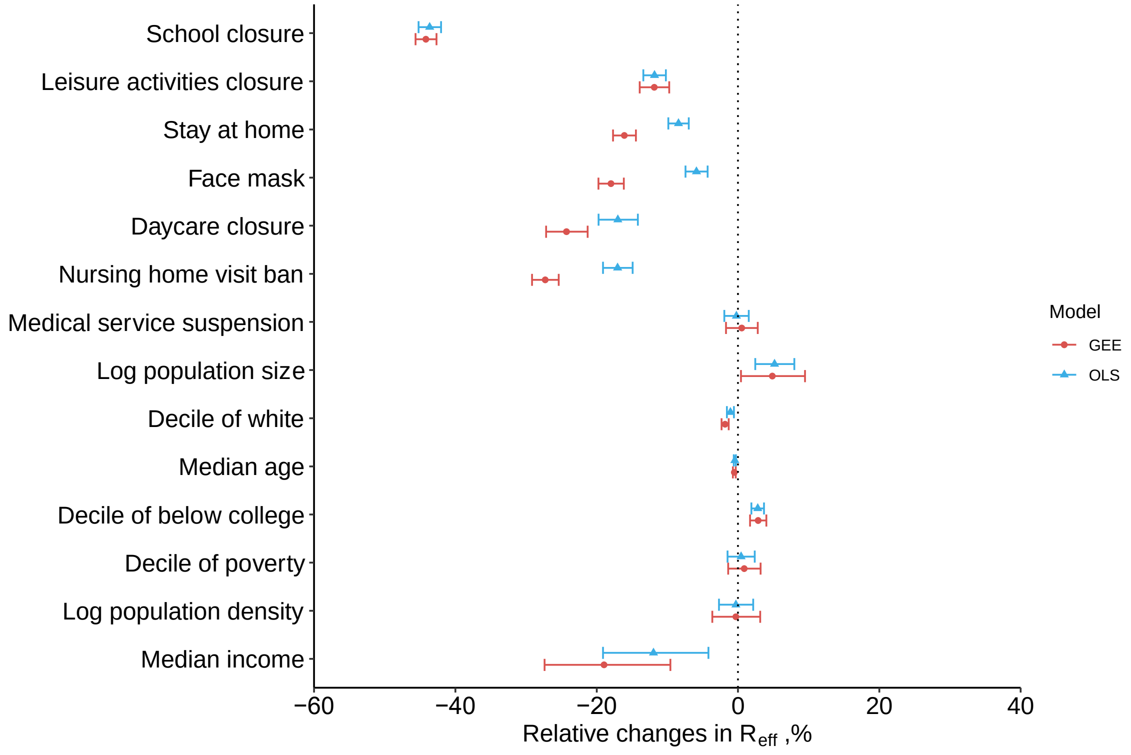


**Supplementary Figure 22. Associations between non-pharmaceutical interventions (NPIs) on transmission, using reproduction numbers ($R_{eff}$) that were estimated from stochastic reconstruction of infections from cases by sampling delay distribution of time from infection to confirmation.** Data are presented as mean and 95% confidence interval for estimates from 32,607 county-weeks. GEEs: generalized estimation equations (red). OLS: ordinary least squares (blue).

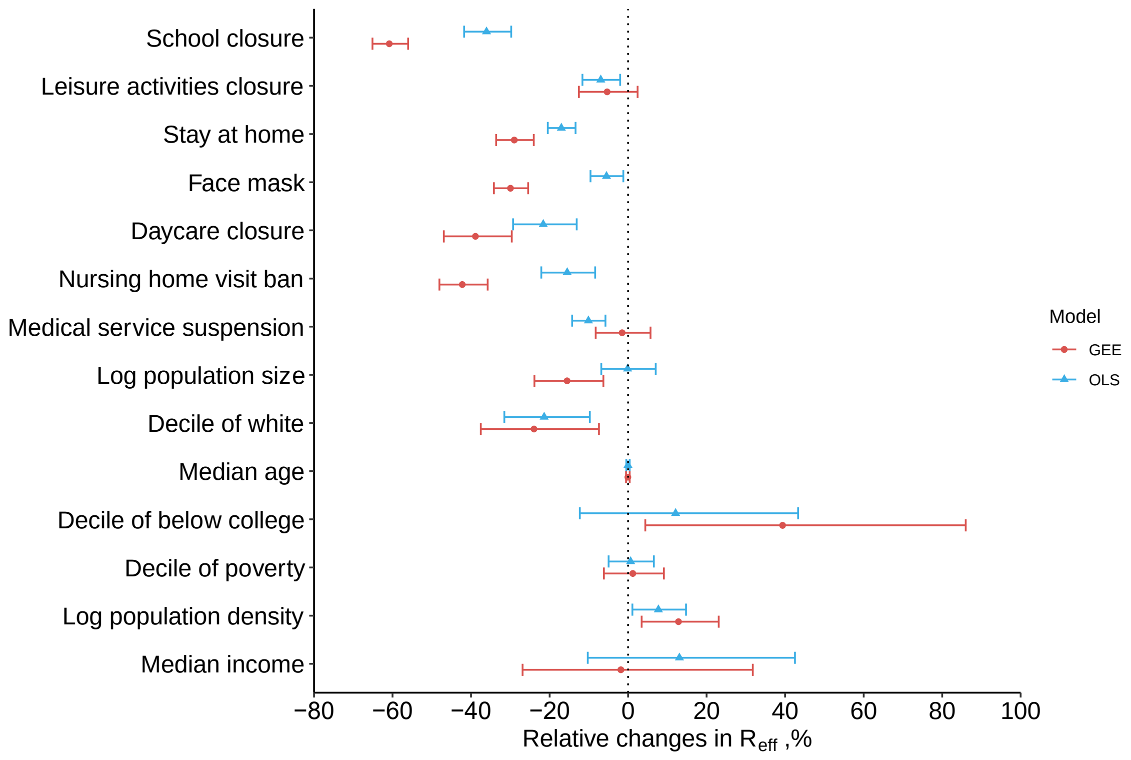


**Supplementary Figure 23. Associations between non-pharmaceutical interventions (NPIs) on transmission, using reproduction numbers ($R_{eff}$) estimated from deconvolution of county-level COVID-19 death reports.** Data are presented as mean and 95% confidence interval for estimates from 19,453 county-weeks. GEEs: generalized estimation equations (red). OLS: ordinary least squares (blue).

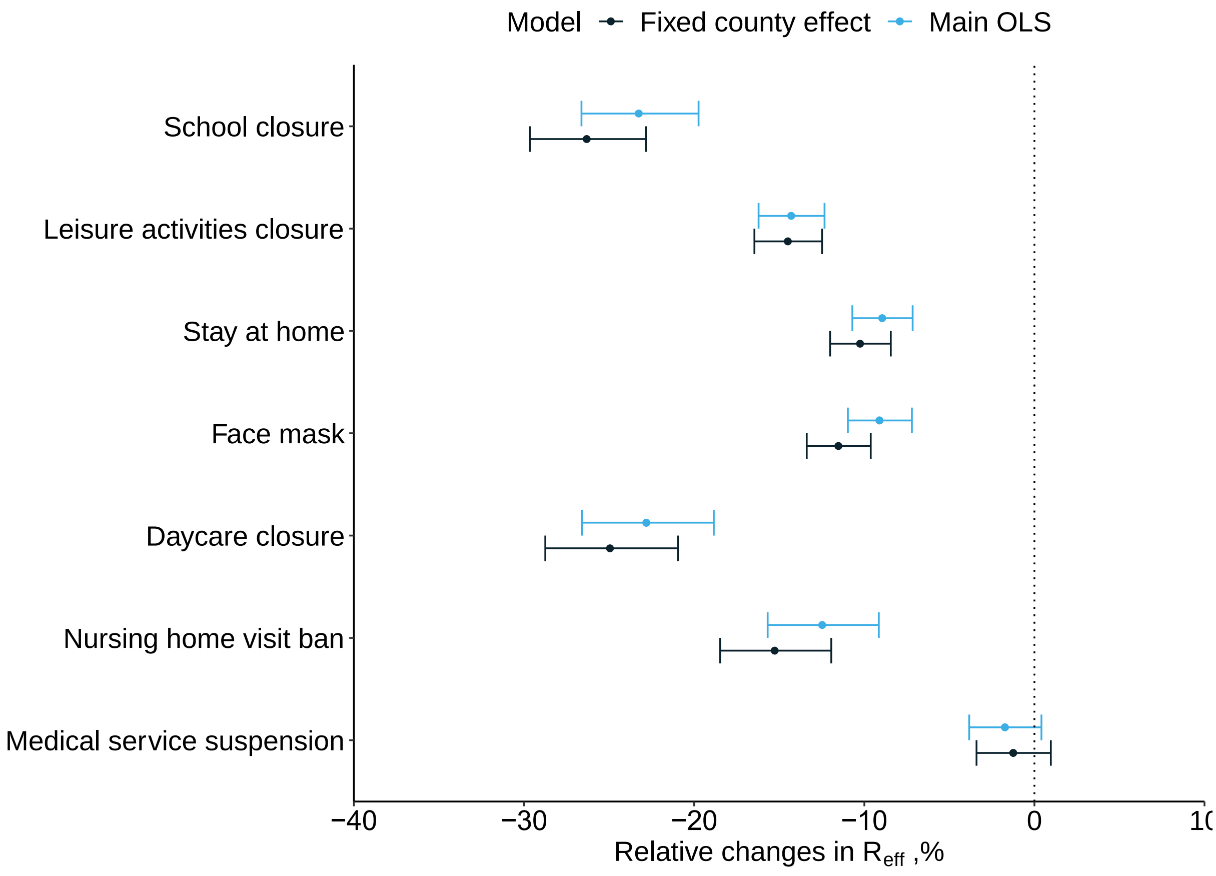


**Supplementary Figure 24. Effect of unobservable county-level population characteristics on the associations between non-pharmaceutical interventions (NPIs) on transmission.** Model was fitted by replacing the county-level characteristics and state-level fixed effects with county-level fixed effects for the main ordinary least squares (OLS) model. Data are presented as mean and 95% confidence interval for estimates from 31,072 county-weeks.

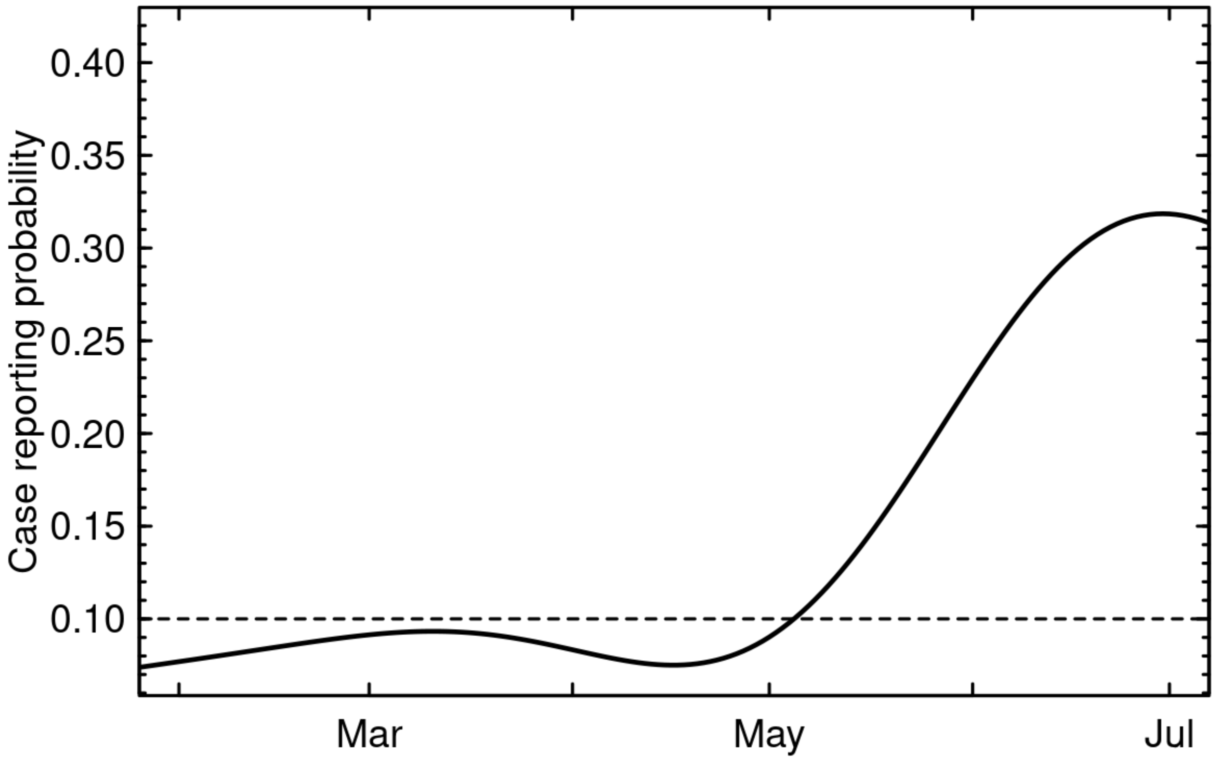


**Supplementary Figure 25. Time-varying case reporting probability used for the analyses in Supplementary Table 7**. The horizontal line shows the case reporting probability assumed in the main model.
