# Supplementary material for: Effect of specific non-pharmaceutical intervention policies on SARS-CoV-2 transmission in the counties of the United States
Source: Nat Commun. 2021 Jun 11;12:3560. doi: 10.1038/s41467-021-23865-8 (PMC8195990; doi:10.1038/s41467-021-23865-8)
Supplement: Supplementary file 2 — Reporting Summary [file 41467_2021_23865_MOESM2_ESM.pdf]

nature research

Corresponding author(s): Derek A. T. Cummings; Carl D. Laird; Justin Lessler

Last updated by author(s): 18-Feb 2021

Reporting Summary

Nature Research wishes to improve the reproducibility of the work that we publish. This form provides structure for consistency and transparency in reporting. For further information on Nature Research policies, see our Editorial Policies and the Editorial Policy Checklist.

Statistics

For all statistical analyses, confirm that the following items are present in the figure legend, table legend, main text, or Methods section.

n/a	Confirmed	
☐	☒	The exact sample size (*n*) for each experimental group/condition, given as a discrete number and unit of measurement
☒	☐	A statement on whether measurements were taken from distinct samples or whether the same sample was measured repeatedly
☐	☒	The statistical test(s) used AND whether they are one- or two-sided<br>*Only common tests should be described solely by name; describe more complex techniques in the Methods section.*
☐	☒	A description of all covariates tested
☐	☒	A description of any assumptions or corrections, such as tests of normality and adjustment for multiple comparisons
☐	☒	A full description of the statistical parameters including central tendency (e.g. means) or other basic estimates (e.g. regression coefficient) AND variation (e.g. standard deviation) or associated estimates of uncertainty (e.g. confidence intervals)
☒	☐	For null hypothesis testing, the test statistic (e.g. *F*, *t*, *r*) with confidence intervals, effect sizes, degrees of freedom and *P* value noted<br>*Give P values as exact values whenever suitable.*
☒	☐	For Bayesian analysis, information on the choice of priors and Markov chain Monte Carlo settings
☐	☒	For hierarchical and complex designs, identification of the appropriate level for tests and full reporting of outcomes
☐	☒	Estimates of effect sizes (e.g. Cohen's *d*, Pearson's *r*), indicating how they were calculated

Our web collection on statistics for biologists contains articles on many of the points above.

Software and code

Policy information about availability of computer code

Data collection	Data on county-level characteristics were obtained using "tidycensus" package version 0.9.9.2 in R (version 4.0.2).
Data analysis	All statistical analyses and data visualizations were performed with R (version 4.0.2) unless particularly specified. Back projection of the reported incidence was performed with the "surveillance" package (version 1.18.0). Inference on effective reproduction numbers were performed with epi_inference (to be released), Pyomo21 (version 5.7.3) and IPOPT in Python. Generalized equation estimations (GEEs) was performed with "geepack" package (version 1.3.1). LASSO was performed with "glmnet" package (version 4.0.2). Mediation analyses for OLS models was performed with "mediation" package (version 4.5.0). Cluster-robust standard errors for OLS models were performed with "fixest" package (version 0.8.3).

For manuscripts utilizing custom algorithms or software that are central to the research but not yet described in published literature, software must be made available to editors and reviewers. We strongly encourage code deposition in a community repository (e.g. GitHub). See the Nature Research guidelines for submitting code & software for further information.

Data

Policy information about availability of data

All manuscripts must include a data availability statement. This statement should provide the following information, where applicable:

- Accession codes, unique identifiers, or web links for publicly available datasets
- A list of figures that have associated raw data
- A description of any restrictions on data availability

We collected the closure time of public schools (K-12) of each county, which can be accessed at https://github.com/UF-IDD/US_County_Rt/blob/main/data/school.csv. Data on other state-level interventions were obtained from an external data set COVID-19 US State Policy Database (CUSP) on July 6, 2020 at https://docs.google.com/spreadsheets/u/1/d/1zu9qEWI8PsOI_i8nI_S29HDGHIIp2lfVMsGxpQ5tvAQ/edit#gid=973655443. Laboratory-confirmed COVID-19 cases and deaths

in the counties of the United States were retrieved from USAFacts (https://usafacts.org/visualizations/coronavirus-covid-19-spread-map/) on July 5, 2020. Data on commuting between counties before the pandemic was obtained from Census Bureau (https://www.census.gov/topics/employment/commuting.html). The authors declare that all data generated or analyzed during this study are made available at https://github.com/UF-IDD/US_County_Rt.

Field-specific reporting

Please select the one below that is the best fit for your research. If you are not sure, read the appropriate sections before making your selection.

- [x] Life sciences
- [ ] Behavioural & social sciences
- [ ] Ecological, evolutionary & environmental sciences

For a reference copy of the document with all sections, see nature.com/documents/nr-reporting-summary-flat.pdf

Life sciences study design

All studies must disclose on these points even when the disclosure is negative.

Sample size	The study was based on daily confirmed COVID-019 cases for each county in the US and the resulting estimated reproduction number effective reproduction numbers. No sample size calculation was needed.
Data exclusions	We excluded county-week when the two-week average case number was zero because robust effective reproduction numbers can not be estimated.
Replication	We confirmed replications for all analyses using the data and codes provided at https://github.com/UF-IDD/US_County_Rt.
Randomization	This is an observational study and randomization is not applicable.
Blinding	This is an observational study and blinding is not applicable.

Reporting for specific materials, systems and methods

We require information from authors about some types of materials, experimental systems and methods used in many studies. Here, indicate whether each material, system or method listed is relevant to your study. If you are not sure if a list item applies to your research, read the appropriate section before selecting a response.

Materials & experimental systems

n/a	Involved in the study	
☒	☐	Antibodies
☒	☐	Eukaryotic cell lines
☒	☐	Palaeontology and archaeology
☒	☐	Animals and other organisms
☒	☐	Human research participants
☒	☐	Clinical data
☒	☐	Dual use research of concern

Methods

n/a	Involved in the study	
☒	☐	ChIP-seq
☒	☐	Flow cytometry
☒	☐	MRI-based neuroimaging
